# Supplementary material for: Spatial coding dysfunction and network instability in the aging medial entorhinal cortex
Source: Nat Commun. 2025 Oct 3;16:8770. doi: 10.1038/s41467-025-63229-0 (PMC12494969; doi:10.1038/s41467-025-63229-0)
Supplement: Supplementary file 1 — Supplementary Information [file 41467_2025_63229_MOESM1_ESM.pdf]

Supplementary Figures & Legends

Spatial Coding Dysfunction and Network Instability in the Aging Medial Entorhinal Cortex

Charlotte S. Herber, Karishma J.B. Pratt, Jeremy M. Shea, Saul A. Villeda, and Lisa M. Giocomo

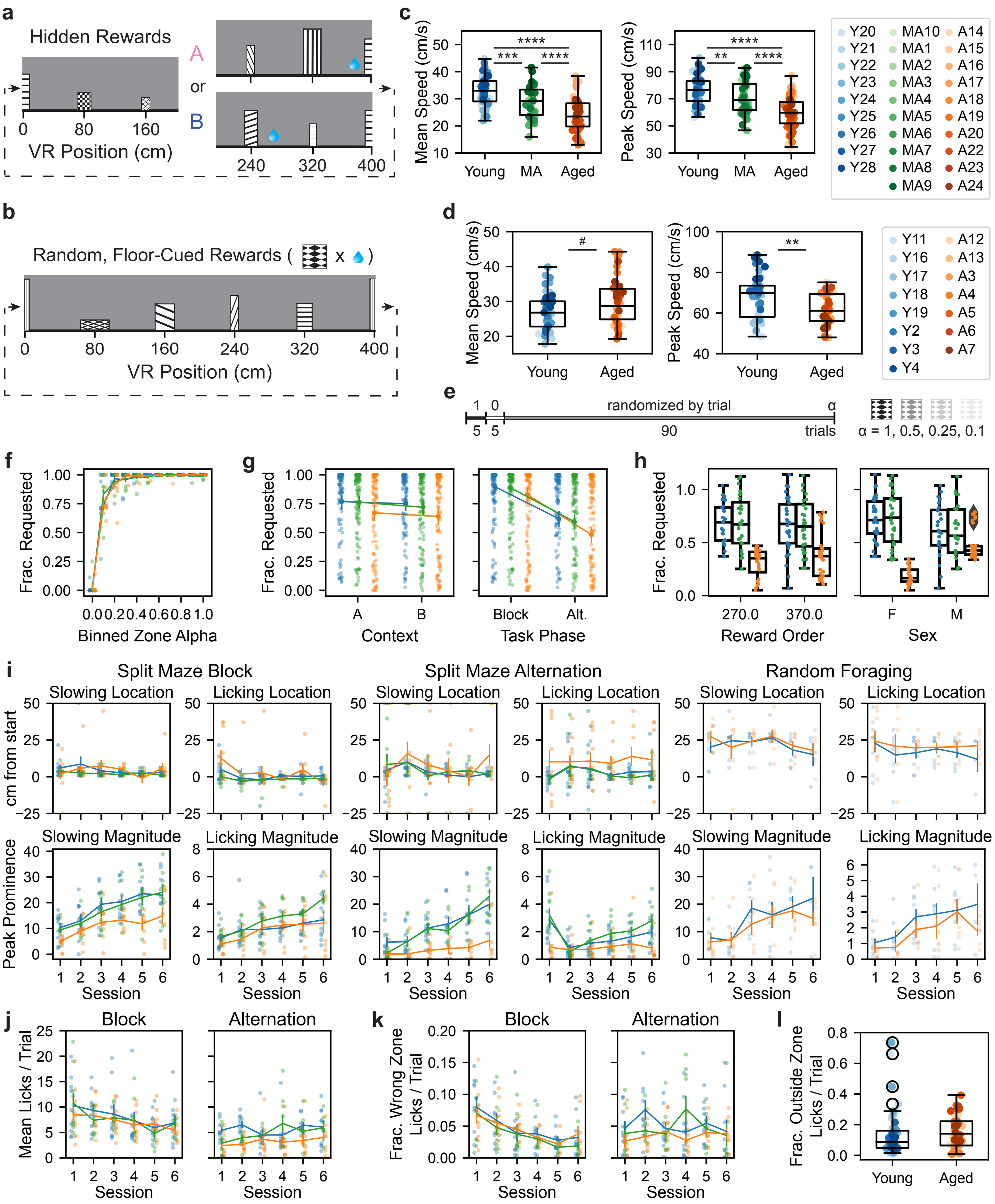
a
Hidden Rewards
A
or
B
0
80
160
VR Position (cm)
240
320
400
b
Random, Floor-Cued Rewards (x)
0
80
160
240
320
400
VR Position (cm)
c
Mean Speed (cm/s)
Young
MA
Aged
Peak Speed (cm/s)
Young
MA
Aged
Y20 Y21 Y22 Y23 Y24 Y25 Y26 Y27 Y28
MA10 MA1 MA2 MA3 MA4 MA5 MA6 MA7 MA8 MA9
A14 A15 A16 A17 A18 A19 A20 A22 A23 A24
d
Mean Speed (cm/s)
Young
Aged
Peak Speed (cm/s)
Young
Aged
Y11 Y16 Y17 Y18 Y19 Y2 Y3 Y4
A12 A13 A3 A4 A5 A6 A7
e
1
0
randomized by trial
α
5
5
90
trials
α = 1, 0.5, 0.25, 0.1
f
Frac. Requested
Binned Zone Alpha
g
Frac. Requested
A
B
Context
Block
Alt.
Task Phase
h
Frac. Requested
270.0
370.0
Reward Order
F
M
Sex
i
Split Maze Block
Split Maze Alternation
Random Foraging
Slowing Location
Licking Location
cm from start
Slowing Magnitude
Licking Magnitude
Peak Prominence
Session
j
Block
Alternation
Mean Licks / Trial
Session
k
Block
Alternation
Frac. Wrong Zone Licks / Trial
Session
l
Frac. Outside Zone Licks / Trial
Young
Aged

Supplementary Fig. 1. Aging did not impair visual contrast sensitivity or reward-triggered licking in VR.

(a) Schematized Split Maze (SM) VR track. **(b)** Schematized Random Foraging (RF) VR track. **(c)** Plotted as in Fig. 2d, mean (left) and peak session running speed (right) across SM age groups (n = 54 young, 55 MA, 54 aged sessions). Running speed decreased with age (mean speed [cm/s]: young vs. MA vs. aged, 32.88 ± 0.73 vs. 28.99 ± 0.76 vs. 23.98 ± 0.86, Kruskal-Wallis test, H = 44.71, $p < 0.0001$, post-hoc Conover test, young vs. MA, p = 0.0004, young vs. aged, $p < 0.0001$; MA vs. aged, p = 0.0001; peak speed [cm/s]: 76.58 ± 1.47 vs. 71.08 ± 1.59 vs. 59.52 ± 1.62, H = 43.53, $p < 0.0001$, post-hoc Conover test, young vs. MA, p = 0.0101; young vs. aged, $p < 0.0001$; MA vs. aged, $p < 0.0001$). Individual dots represent session averages colored by age group and mouse identity. Dot colors maintained in Fig. 2d and Supplementary Figs. 3d, 3b, 5d, and 5j. **(d)** As in (c) across age groups in the RF task (n = 43 young vs. 42 aged sessions). Mean speed was unchanged across age groups in the RF task (young vs. aged, 27.19 ± 0.8 vs. 29.96 ± 1.06, Wilcoxon rank sum test, p = 0.087), but peak running speed (right) was greater among young sessions (68.14 ± 1.58 vs. 61.72 ± 1.19, p = 0.0036). Individual dots represent session averages colored by age group and mouse identity. **(e)** Schematized visual contrast sensitivity assessment trial structure where reward zone opacity is given by alpha (α = 0 if invisible vs. α = 1 if opaque) (left). Sample reward zones at four different alpha values (right). **(f)** Binned visual contrast sensitivity assessment performance for SM mice (n = 9 young, 10 MA, 10 aged) (bin size = 0.1). Mouse visual contrast sensitivity threshold, estimated as the center of the psychometric curve fitted to their task performance (see Methods), did not differ across age groups (center: young vs. MA vs. aged, 0.0981 ± 0.0007 vs. 0.0973 ± 0.0005 vs. 0.0975 ± 0.0004, Kruskal-Wallis test, H = 1.508, p = 0.4704). The highest estimated individual contrast sensitivity threshold was 0.102, indicating all SM mice could resolve higher contrast visual cues. Individual animals are plotted as pale dots jittered by age order. **(g)** Interaction of context identity (left, A vs. B) and task phase (right, block vs. alternation) with age effects on SM task performance (fraction [frac.] of reward requested) across trial blocks (A, B, A`, B` trials), as fitted by LMM. Context B and alternation trial structure both negatively predicted task performance in all age groups (B vs. A, β = -0.154, $p < 0.0001$; Aged x B vs. Aged x A, β = 0.002, p = 0.96; Alternation [Alt.] vs. Block, β = -0.593, $p < 0.0001$; Aged x Alt. vs. Aged x Block, β = -0.027, p = 0.48). Bold lines and dots indicate age group means with age group SEM indicated by vertical bars. Dots represent LMM-fitted performance for sessions trial blocks, colored and jittered by age group. **(h)** Box and whisker plot of the interaction of reward order (left; 270 vs. 370 cm context A reward location) and sex (right; female [F] vs. male [M]) with age effects on SM alternation performance (fraction [frac.] of reward requested), as fitted by LMM. Reward order did not predict performance overall (β = -0.046, p = 0.673) or among aged mice (270 x Aged vs. 370 x Aged: β = 0.118, p = 0.455; interaction effect omitted from subsequent models). This was also true for the effect of reward order on block phase performance (not shown; β = 0.012, p = 0.699; interaction effect omitted from final model). Male sex did not predict alternation performance (Male vs. Female, β = -0.101, p = 0.340), but male sex positively predicted aged performance (Aged x Male vs. Aged x Female, β = 0.371, p = 0.015). Age-sex effects on block performance were less apparent (not shown; Male vs. Female, β = -0.038, p = 0.489; Aged Male vs. Aged Female, β = 0.136, p = 0.111). Dots represent LMM-fitted performance for individual sessions, colored by age group. **(i)** Peak slowing location (top left) and magnitude (bottom left) and peak licking location (top right) and magnitude (bottom right), relative to the start of each reward zone, averaged across trials in each session, for each SM task phase (block, left; alternation, middle) and in the RF task (right). Earlier licking and slowing indicates greater reward anticipation, whereas greater prominence of the peak of mean reward-triggered slowing and licking behavior indicates greater behavioral stereotypy. In the SM block phase, LMMs showed that age predicts later licking (top right) but not slowing (top left) (Block Licking Location: Aged vs. Young, β = 13.964, p = 0.003; Slowing Location: Aged vs. Young, p = 0.391). SM block mean reward-triggered slowing and licking stereotypy did not differ by age group and increased over days (Slowing Magnitude: Aged vs. Young, p = 0.451; Session, β = 2.737, $p < 0.0001$; Licking Magnitude: Aged vs. Young, p = 0.375; Session, β = 0.232, p = 0.003). Aged mice improved SM block slowing stereotypy less than young mice over days (Slowing Magnitude: Session x Aged, β = -1.205, p = 0.049; Licking Magnitude: Session x Aged, p = 0.652). In the SM alternation phase, slowing location did not differ with age or across sessions (Aged vs. Young, p = 0.218; Session, p = 0.657; Session x Aged, p = 0.409). The LMM of SM alternation licking location did not converge. As in the SM block phase, slowing magnitude improved less over sessions for aged mice (Aged vs. Young, p = 0.274; Session, β = 2.844, $p < 0.0001$; Session x Aged, β = -2.050, $p < 0.0001$). SM alternation licking magnitude was lower in aged mice but invariant over sessions (Aged vs. Young, β = -1.484, p = 0.019; Session, p = 0.557; Session x Aged, p = 0.656). In the RF task, reward-triggered maximal slowing (top left) and licking (top right) location did not differ over sessions or between

age groups (Slowing Location: Aged vs. Young, $p = 0.235$; Session, $p = 0.375$; Licking Location: Aged vs. Young, $p = 0.332$; Session, $p = 0.264$). The prominence of peak RF reward-triggered slowing (bottom left) and licking (bottom right) did not differ across age groups and improved equivalently over sessions across age groups (Slowing Magnitude: Aged vs. Young, $p = 0.755$; Session, $\beta = 3.049$, $p < 0.0001$, Session x Aged, $p = 0.388$; Licking Magnitude: Aged vs. Young, $p = 0.645$; Session, $\beta = 0.513$, $p < 0.0001$; Session x Aged, $p = 0.333$). These results indicate that aged reward-triggered licking behavior was uniquely impaired the SM alternation task, while SM block and RF task licking behavior stereotypy was not age-modulated and improved over sessions for all age groups. Individual session data are plotted as dots, colored and jittered by age group. Bold lines represent age group means with age group SEM indicated by vertical bars. **(j)** Mean non-consummatory licks per trial by SM task phase over sessions across age groups, plotted as in (i). Session negatively predicted SM block lick rate but not alternation lick rate via LMM (Block: Session, $\beta = -0.812$, $p = 0.002$; Alternation: Session, $p = 0.832$). Being aged negatively predicted alternation but not block lick rate (Block: Aged vs. Young, $p = 0.154$; Alternation: Aged vs. Young, $\beta = -3.974$, $p = 0.006$), confirming that aged mice fail during this task phase by missing rewards. **(k)** As in (j), mean fraction (frac.) licks in the opposite (wrong) reward zone per trial as a measure of Type 1 lick error rate. Session negatively predicted SM block but not alternation lick error rate (Block: Session, $\beta = -0.009$, $p < 0.0001$; Alternation: Session, $p = 0.182$). Being aged predicted neither SM alternation nor block lick error rate (Block: Aged vs. Young, $p = 0.429$; Alternation: Aged vs. Young, $p = 0.068$). **(l)** The mean fraction (frac.) of trial licks outside the reward zone in the RF task, plotted as in (d), as a measure of Type 1 error rate ($n = 43$ young vs. 42 aged sessions). Lick error rate did not differ across age groups (young vs. aged, 0.14 ± 0.02 vs. 0.16 ± 0.02, Wilcoxon rank sum test, $p = 0.1759$). This suggests that Type 1 lick error rates were low in VR in the SM and RF tasks across age groups, consistent with the high energetic penalty of halting the running wheel. Related to Fig. 1.

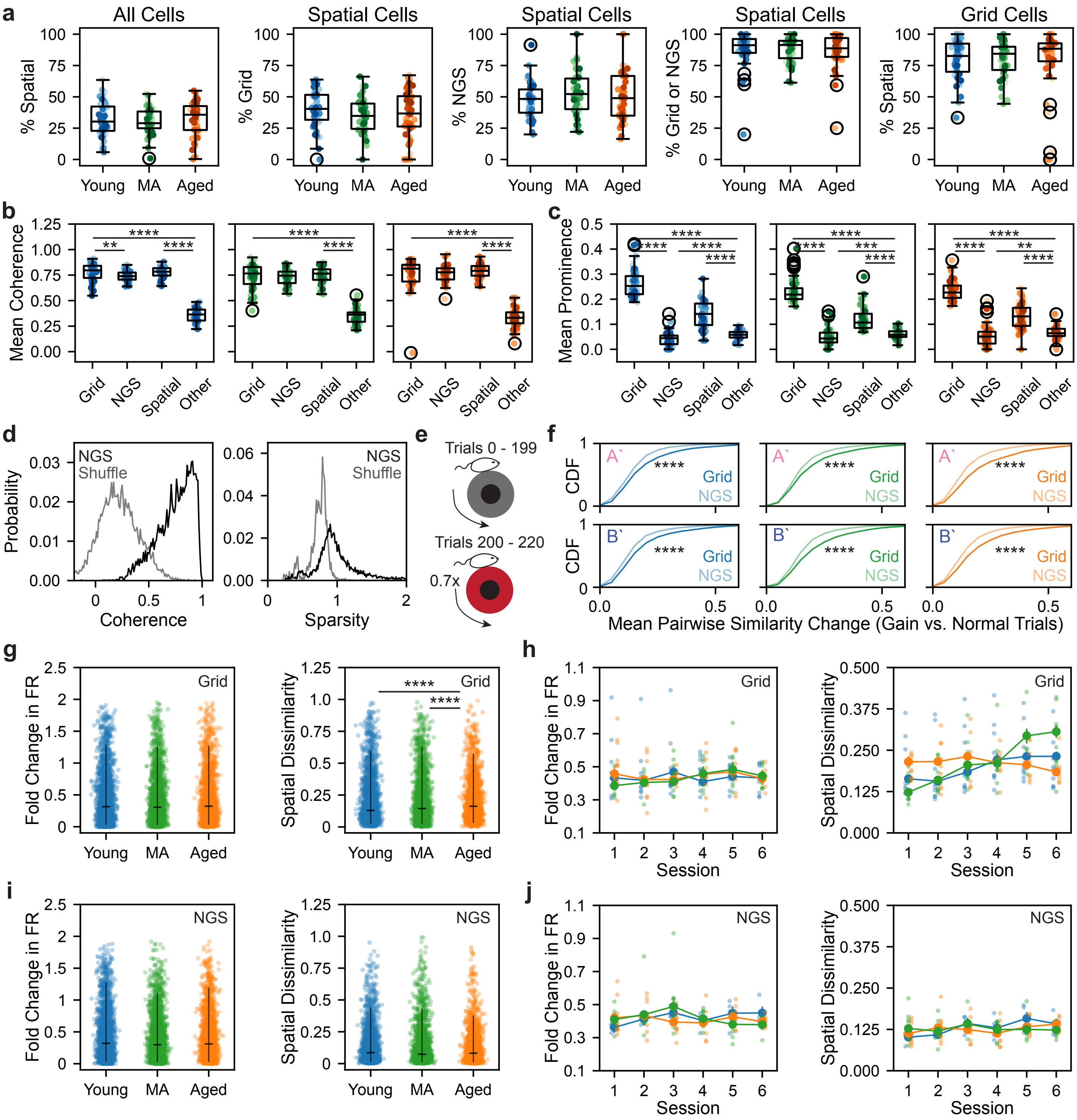

a
All Cells
Spatial Cells
Spatial Cells
Spatial Cells
Grid Cells
% Spatial
% Grid
% NGS
% Grid or NGS
% Spatial
Young
MA
Aged
b
Mean Coherence
Grid
NGS
Spatial
Other
c
Mean Prominence
d
NGS
Shuffle
Probability
Coherence
Sparsity
e
Trials 0 - 199
Trials 200 - 220
0.7x
f
A`
B`
CDF
Grid
NGS
Mean Pairwise Similarity Change (Gain vs. Normal Trials)
g
Fold Change in FR
Spatial Dissimilarity
h
Session
i
j

Supplementary Fig. 2. Grid cells exhibit stronger responses to VR gain changes and context-driven global remapping than NGS cells across age groups.

(a) Box and whisker plots, plotted as in Fig. 2d, of the density of spatially tuned (spatial) cells among all recorded cells (first); of distance-tuned grid cells among spatial cells (second); of non-grid spatial (NGS) cells among spatial cells (third); of grid or NGS cells among spatial cells (fourth); and of spatial cells among grid cells (fifth), by session across age groups (n = 54 young, 58 MA, and 55 aged sessions with spatial cells). The spatial cell density did not differ across age groups (% spatial, young vs. MA vs. aged, 31.90 ± 1.86 vs. 30.32 ± 1.30 vs. 31.62 ± 1.91, Kruskal-Wallis H-test, H = 1.11, p = 0.58). Neither did grid cell density among spatial cells (% grid, young vs. MA vs. aged, 40.56 ± 1.97 vs. 35.22 ± 1.73 vs. 36.76 ± 2.46, Kruskal-Wallis H-test, H = 4.09, p = 0.13) or NGS cell density among spatial cells (47.59 ± 1.86 vs. 52.29 ± 2.14 vs. 50.49 ± 2.67, Kruskal-Wallis H-test, H = 2.65, p = 0.27). Most spatial cells also met grid or NGS cells criteria, and there was no age difference in the density of grid or non-grid spatial cells (% grid or NGS, young vs. MA vs. aged, 88.16 ± 1.75 vs. 87.50 ± 1.24 vs. 87.24 ± 1.76, Kruskal-Wallis H-test, H = 0.82, p = 0.66). Finally, most grid cells also met spatial cell criteria, to an equivalent extent across age groups (n = 53 young, 57 MA, and 53 aged sessions with grid cells; % spatial, young vs. MA vs. aged, 80.31 ± 2.08 vs. 80.42 ± 1.83 vs. 80.37 ± 3.16, Kruskal-Wallis H-test, H = 1.96, p = 0.38). This confirms that grid and non-grid spatial cells were mutually exclusive subsets of spatially tuned SM cells with comparable density across age groups. **(b)** Plotted as in (a), the mean spatial firing coherence of grid, non-grid spatial (NGS), spatial, and all other excitatory cells in each session by age group (n = 53 young [left], 57 MA [middle], 53 aged [right] sessions with grid cells; n = 54 young [left], 58 MA [middle], and 55 aged [right] sessions with NGS, spatial, and other cells). Among young sessions, mean grid, NGS, and spatial cell coherence each exceeded that of other excitatory cells (grid vs. NGS vs. spatial vs. other, 0.7743 ± 0.0123 vs. 0.7428 ± 0.0071 vs. 0.7768 ± 0.0077 vs. 0.3571 ± 0.009; post-hoc Conover test: grid vs. other, p < 0.0001; NGS vs. other, p < 0.0001; spatial vs. other, p < 0.0001). Young grid cells had greater spatial coherence than young NGS cells (post-hoc Conover test: grid vs. NGS, p = 0.0013). Among MA sessions, mean grid and NGS cell spatial coherence was equivalent, and mean grid, NGS, and spatial cell coherence each exceeded that of other excitatory cells (grid vs. NGS vs. spatial vs. other, 0.7383 ± 0.0153 vs. 0.731 ± 0.0103 vs. 0.7512 ± 0.0102 vs. 0.3488 ± 0.0093; post-hoc Conover test: grid vs. NGS, p = 0.40; grid vs. other, p < 0.0001; NGS vs. other, p < 0.0001; spatial vs. other, p < 0.0001). This was also true among aged sessions (grid vs. NGS vs. spatial vs. other, 0.7633 ± 0.0198 vs. 0.7611 ± 0.0108 vs. 0.7842 ± 0.0099 vs. 0.3315 ± 0.0119; post-hoc Conover test: grid vs. NGS, p = 0.34; grid vs. other, p < 0.0001; NGS vs. other, p < 0.0001; spatial vs. other, p < 0.0001). This indicates that, generally, classified SM grid and NGS cells were comparably and strongly spatially tuned. **(c)** As in (b) for the mean dark firing rate autocorrelation peak prominence, as a measure of distance-tuning strength, of co-recorded cells of each type by age group. Among young sessions, the mean peak prominence of grid cells exceeded that of NGS and other, non-spatial excitatory cells (grid vs. NGS vs. spatial vs. other, 0.2616 ± 0.0074 vs. 0.0407 ± 0.0036 vs. 0.1398 ± 0.007 vs. 0.0574 ± 0.0023; post-hoc Conover test: grid vs. NGS, p < 0.0001; grid vs. other, p < 0.0001; NGS vs. other, p < 0.0001; spatial vs. other, p < 0.0001). Among MA sessions, the mean peak prominence of grid cells also exceeded that of NGS and other, non-spatial excitatory cells (grid vs. NGS vs. spatial vs. other, 0.2322 ± 0.0066 vs. 0.0477 ± 0.0044 vs. 0.1235 ± 0.0065 vs. 0.0607 ± 0.0022; post-hoc Conover test: grid vs. NGS, p < 0.0001; grid vs. other, p < 0.0001; NGS vs. other, p = 0.0010; spatial vs. other, p < 0.0001). Among aged sessions, the mean peak prominence of grid cells also exceeded that of NGS and other, non-spatial excitatory cells (grid vs. NGS vs. spatial, 0.2338 ± 0.0054 vs. 0.0539 ± 0.0057 vs. 0.1332 ± 0.0068 vs. 0.0657 ± 0.0032; post-hoc Conover test: grid vs. NGS, p < 0.0001; grid vs. other, p < 0.0001; NGS vs. other, p = 0.0082; spatial vs. other, p < 0.0001). These results confirm that grid cells constituted the most strongly distance-tuned subset of MEC excitatory cells in each age group. In each age group, mean spatial cell distance tuning strength was intermediate compared to that of grid and NGS cells, reflecting that spatial cells constitute a mixture of grid and NGS cells. **(d)** Probability density of the spatial firing coherence (left) and sparsity (right) of NGS cell activity on Context A SM block trials vs. shuffle activity. The spatial firing coherence and sparsity of classified NGS cells' activity significantly exceeds that of their shuffled activity, pooling cells across age groups (n = 6,559 model pairs; coherence, NGS vs. shuffle, 0.74 ± 0.002 vs. 0.22 ± 0.002, Wilcoxon signed-rank, p < 0.0001; sparsity, NGS vs. shuffle, 1.1 ± 0.009 vs. 0.72 ± 0.002, p < 0.0001). The average 99th percentile of shuffle coherence and sparsity scores were 0.4705 ± 0.0009 and 1.3739 ± 0.0118 (n = 43,388 total SM cells). Bin sizes were 0.01. **(e)** Schematized SM task gain manipulation, indicating 200 trials with a fixed relationship between visual and motor feedback (top, normal trials) and 20 trials that required the animal to run 1.42x as far to travel the same VR distance (bottom, gain

change trials). **(f)** Cumulative density function (CDF) of SM gain change responses, computed as the mean pairwise similarity change across gain change and non-gain change trials (see Methods), in young, MA, and aged (left to right) grid vs. NGS cells on A\` trials (top) and B\` trials (bottom) (n = 2441 young, 2035 MA, 2032 aged grid cells; n = 2154 young, 2347 MA, 2058 aged NGS cells). In each age group, grid cells responded stronger than NGS cells to A\` gain change trials (gain change response, grid vs. NGS cells, Young: 0.19 ± 0.003 vs. 0.14 ± 0.002, Wilcoxon rank sum test, $p < 0.0001$; MA: 0.20 ± 0.003 vs. 0.16 ± 0.002, $p < 0.0001$; Aged: 0.22 ± 0.004 vs. 0.15 ± 0.002, $p < 0.0001$). The same was true for B\` gain responses (Young: 0.19 ± 0.003 vs. 0.14 ± 0.002, $p < 0.0001$; MA: 0.21 ± 0.003 vs. 0.16 ± 0.002, $p < 0.0001$; Aged: 0.20 ± 0.003 vs. 0.14 ± 0.002, $p < 0.0001$). This validates grid vs. NGS cell classification by distance-tuning during dark running. Aged grid cells exhibited stronger gain responses in both contexts (A\`: Aged vs. Young, $\beta = 0.065$, $p = 0.017$; B\`: Aged vs. Young, $\beta = 0.061$, $p = 0.034$). **(g)** Absolute fold change in firing rate (FR) (left) and spatial dissimilarity (right) across Context A and B block trials for grid cells in each age group, as measures of rate and global remapping strength, respectively (n = 2441 young, 2035 MA, 2032 aged grid cells) (horizontal bars, median; vertical bars, 5th to 95th percentile). While grid rate remapping strength did not differ among age groups (young vs. MA vs. aged, 43.29 ± 0.79% vs. 39.66 ± 0.71% vs. 43.93 ± 0.86%; Kruskal-Wallis H test, H = 3.21, $p = 0.20$; post-hoc Conover test: young vs. MA, $p = 0.55$; young vs. aged, $p = 0.55$; aged vs. MA, $p = 0.22$), global remapping strength did (young vs. MA vs. aged, 0.19 ± 0.0037 vs. 0.21 ± 0.0042 vs. 0.21 ± 0.0038; Kruskal-Wallis H test, H = 38.60, $p < 0.0001$; post-hoc Conover test: young vs. MA, $p = 0.14$; young vs. aged, $p < 0.0001$; aged vs. MA, $p < 0.0001$). **(h)** Effect of age and session interaction on the mean grid cell rate remapping strength (left) and global remapping strength (right), fitted by separate LMMs, each plotted as in Fig. 2h. Rate remapping strength decreased modestly over sessions for young and aged grid cells (Session x Young, $\beta = -1.208$, $p = 0.009$; Session x MA, $\beta = 1.699$, $p = 0.001$; Session x Aged, $\beta = -0.642$, $p = 0.306$). By contrast, grid global remapping strength improved only for young and MA grid cells over sessions (Session x Young, $\beta = 0.010$, $p < 0.0001$; Session x MA, $\beta = 0.032$, $p < 0.0001$; Session x Aged, $\beta = -0.006$, $p = 0.036$). **(i)** As in (g), for NGS cells in each age group (n = 2154 young, 2347 MA, 2058 aged NGS cells). Rate remapping strength differed among age groups but not between young and aged NGS cells (left) (young vs. MA vs. aged, 43.71 ± 0.84% vs. 42.23 ± 0.86% vs. 41.74 ± 0.81%; Kruskal-Wallis H test, H = 6.27, $p = 0.043$; post-hoc Conover test: young vs. MA, $p = 0.88$; young vs. aged, $p = 0.68$; aged vs. MA, $p = 0.88$). Global remapping strength differed among age groups but between young and aged cells (right) (young vs. MA vs. aged, 0.13 ± 0.0030 vs. 0.12 ± 0.0030 vs. 0.12 ± 0.0029; Kruskal-Wallis H test, H = 14.21, $p = 0.0008$; post-hoc Conover test: young vs. MA, $p = 0.26$; young vs. aged, $p = 0.31$; aged vs. MA, $p = 0.70$). Rate remapping strength was equivalent across those cell types in young and aged mice (Wilcoxon rank sum test, grid vs. NGS cells: young, $p = 0.73$; MA, $p = 0.33$; aged, $p = 0.14$), while global remapping strength was greater in grid vs. NGS cells (young, $p < 0.0001$; MA, $p < 0.0001$; aged, $p < 0.0001$). **(j)** As in (h), for NGS cells. Rate remapping strength declined for MA and aged NGS cells over sessions (Session x Young, $\beta = 1.439$, $p = 0.024$; Session x MA, $\beta = -1.097$, $p = 0.007$; Session x Aged, $\beta = -0.879$, $p = 0.041$). Global remapping strength improved for only young NGS cells over sessions (Session x Young, $\beta = 0.010$, $p < 0.0001$; Session x MA, $\beta = -0.001$, $p = 0.626$; Session x Aged, $\beta = 0.003$, $p = 0.101$). Related to Fig. 2.

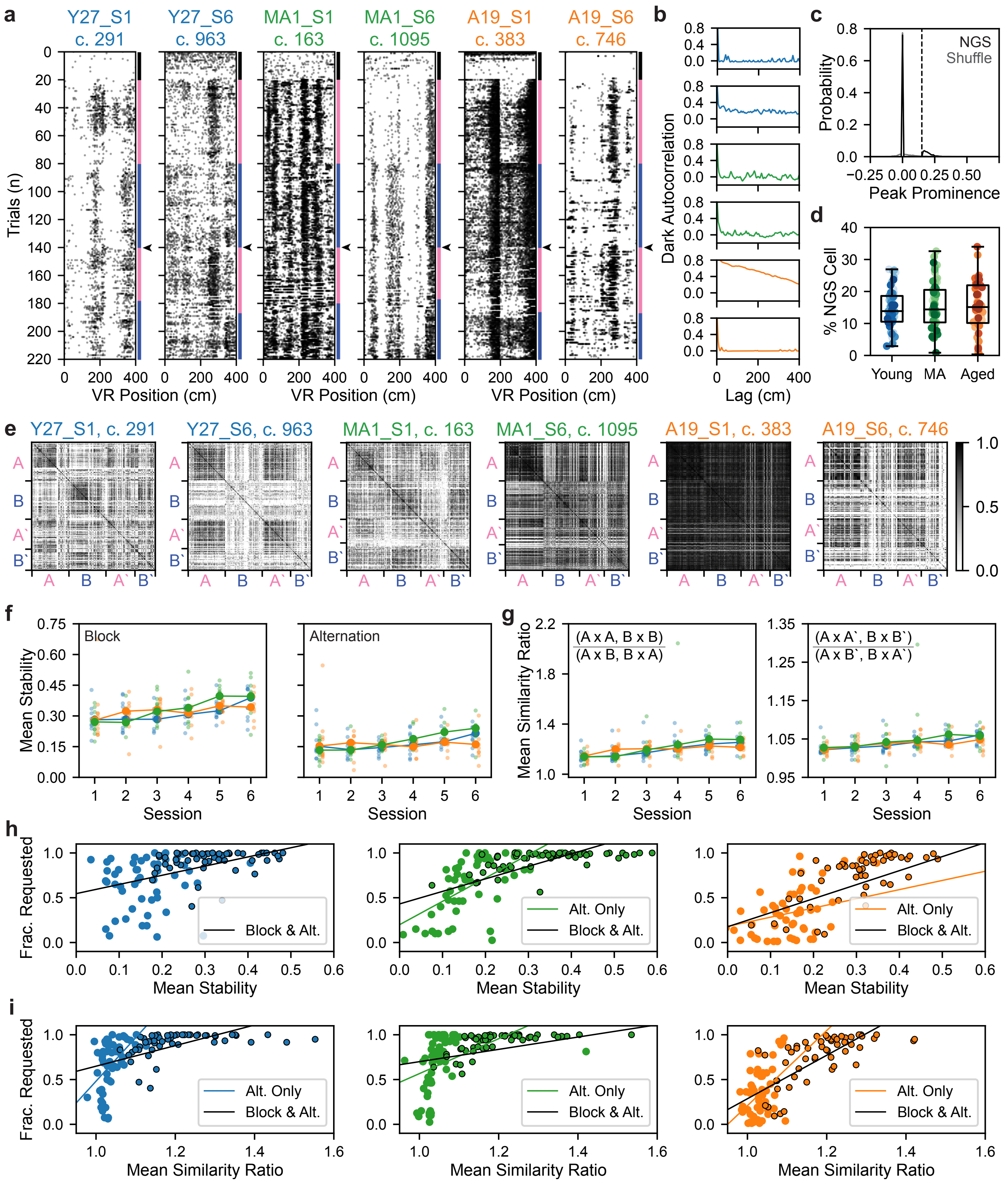

a
Y27_S1 c. 291
Y27_S6 c. 963
MA1_S1 c. 163
MA1_S6 c. 1095
A19_S1 c. 383
A19_S6 c. 746
Trials (n)
VR Position (cm)
b
Dark Autocorrelation
Lag (cm)
c
Probability
NGS
Shuffle
Peak Prominence
d
% NGS Cell
Young
MA
Aged
e
Y27_S1, c. 291
Y27_S6, c. 963
MA1_S1, c. 163
MA1_S6, c. 1095
A19_S1, c. 383
A19_S6, c. 746
A
B
A`
B`
f
Block
Alternation
Mean Stability
Session
g
Mean Similarity Ratio
(A x A, B x B) / (A x B, B x A)
(A x A`, B x B`) / (A x B`, B x A`)
Session
h
Frac. Requested
Mean Stability
Alt. Only
Block & Alt.
i
Frac. Requested
Mean Similarity Ratio
Alt. Only
Block & Alt.

Supplementary Fig. 3: Non-grid spatial cell activity changes less across VR contexts and over days.

(a) As in Fig. 2a, raster plots for NGS cell activity. **(b)** As in Fig. 2b, for NGS cells in (a) revealing a lack of distance tuning. This is indicated by the lack of large peaks in the dark autocorrelation of each cell. **(c)** As in Fig. 2c, for NGS cells. Dark autocorrelation peak prominence from NGS cell vs. shuffle activity differed significantly (n = 6,559 model pairs, mean ± SEM prominence, NGS vs. shuffle activity, 0.047 ± 0.001 vs. 0.011 ± 0.0007, Wilcoxon signed-rank, $p < 0.0001$) (see Methods). However, NGS cell dark autocorrelation peak prominence was significantly lower than that of grid cells (NGS vs. grid activity, 0.047 ± 0.001 vs. 0.25 ± 0.001, Wilcoxon rank sum, $p < 0.0001$). **(d)** As in Fig. 2d, for NGS cells (n = 54 young, 58 MA, 55 aged sessions). NGS cell density by session does not differ among SM age groups (% NGS cells, young vs. MA vs. aged, 14.59 ± 0.87 vs. 15.73 ± 0.95 vs. 15.14 ± 1.08, H = 0.48, p = 0.79). **(e)** As in Fig. 2e, for NGS cells in (a) from left to right. Qualitatively, NGS cells displayed more similar spatial firing across trials and contexts than grid cells. **(f)** As in Fig. 2f for mean NGS cell spatial firing stability during block (left) and alternation (right) task phases (n = 2154 young, 2347 MA, 2058 aged NGS cells). NGS cell stability improved over sessions in each task phase for young and MA mice (Session x Young, Session x MA: Block: $\beta = 0.018$, $\beta = 0.030$; Alt.: $\beta = 0.011$, $\beta = 0.023$) (all $p < 0.0001$), but not aged mice during alternation (Session x Aged, Block: $\beta = 0.006$, p = 0.042; Alt.: $\beta = -0.001$, p = 0.540). **(g)** As in Fig. 2g and (f) for mean NGS cell similarity ratio by task phase. NGS cell similarity ratio improved over sessions in each task phase for young and MA mice (Session x Young, Session x MA: Block: $\beta = 0.024$, $\beta = 0.031$; Alt.: $\beta = 0.007$, $\beta = 0.007$) (all $p < 0.0001$), but not aged mice (Session x Aged: Block: $\beta = 0.006$, p = 0.089). Aged NGS cell mean similarity improved less during alternation vs. younger counterparts (Session x Aged: Alt.: $\beta = 0.004$, p = 0.002). **(h)** As in Fig. 2h for task performance (fraction [frac.] reward requested) vs. mean NGS cell stability for each age group, splitting sessions by task phase (n = 108 young [left], 116 MA [middle], and 110 aged [right] task phases). In each age group, task performance related to NGS cell stability across phases (Young, r = 0.45; MA, r = 0.65; Aged, r = 0.66) (all $p < 0.0001$). This was also true for MA and aged NGS cells considering alternation alone (Young, p = 0.19; MA, r = 0.64, $p < 0.0001$; Aged, r = 0.42, p = 0.0020). **(i)** As in Fig. 2i and **(h)** for mean NGS cell similarity ratio. This related to performance across phases (Young, r = 0.53; MA, r = 0.42; Aged, r = 0.74) (all $p < 0.0001$) and during alternation alone (Young, r = 0.49, p = 0.0002; MA, r = 0.36, p = 0.0054; Aged, r = 0.43, p = 0.0011) in all age groups. Related to Fig. 2.

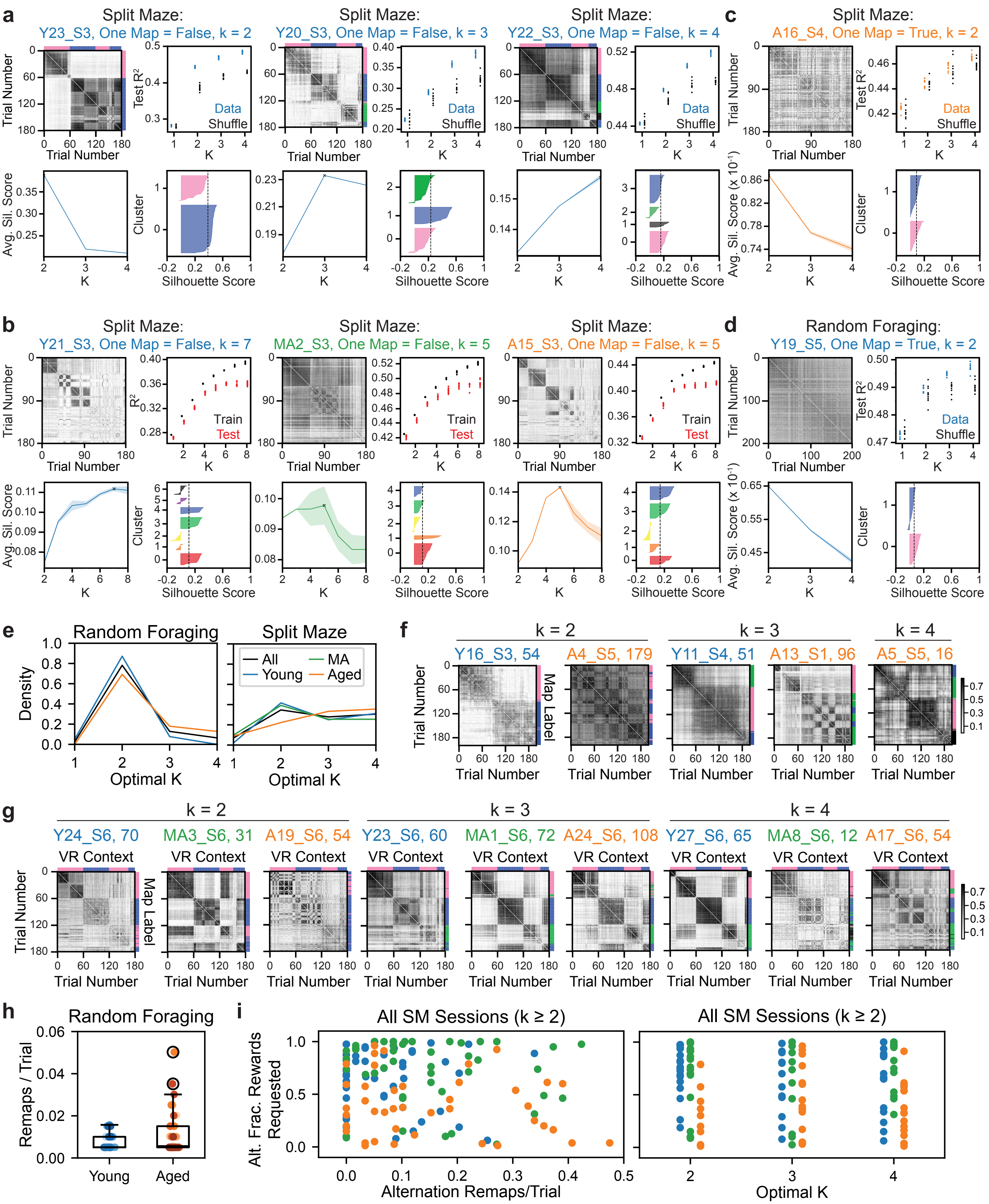
a
Split Maze:
Y23_S3, One Map = False, k = 2
Y20_S3, One Map = False, k = 3
Y22_S3, One Map = False, k = 4
Trial Number
Test R²
Data
Shuffle
K
Avg. Sil. Score
Cluster
Silhouette Score
c
A16_S4, One Map = True, k = 2
Avg. Sil. Score (x 10⁻¹)
b
Y21_S3, One Map = False, k = 7
MA2_S3, One Map = False, k = 5
A15_S3, One Map = False, k = 5
R²
Train
Test
d
Random Foraging:
Y19_S5, One Map = True, k = 2
e
Random Foraging
Split Maze
Density
All
Young
MA
Aged
Optimal K
f
k = 2
k = 3
k = 4
Y16_S3, 54
A4_S5, 179
Y11_S4, 51
A13_S1, 96
A5_S5, 16
Map Label
g
Y24_S6, 70
MA3_S6, 31
A19_S6, 54
Y23_S6, 60
MA1_S6, 72
A24_S6, 108
Y27_S6, 65
MA8_S6, 12
A17_S6, 54
VR Context
h
Remaps / Trial
Young
Aged
i
All SM Sessions (k ≥ 2)
Alt. Frac. Rewards Requested
Alternation Remaps/Trial

Supplementary Fig. 4: K-means clustering with k optimization identified grid network spatial maps.

(a) Illustrated k hyperparameter optimization procedure for example young SM sessions fit by k-means models with optimal k = 2, 3, and 4 (from left to right) (see Methods). Subpanel titles indicate mouse and session (S) number, if optimal k was greater than 1, and optimal k. Y-axes are shared across subpanels. The VR context (top x-axis) and k-means-labeled (right y-axis) trial-by-trial grid network similarity matrix using optimal k (top left) and 10-fold cross-validation test R^2 for each possible k for real vs. shuffled firing rate tensors (top right), and average (avg.) silhouette (sil.) score for possible k ≥ 2 on those 10 repetitions (bottom left; SEM shaded) are shown. The optimal k-means model captured similarity matrix structure (top left), yielded significantly greater test R^2 than when applied to shuffle data (top right), and maximized the average silhouette score (bottom left). Furthermore, it produced high quality clusters with distributions of trial silhouette scores that intersected the average silhouette score (black dashed vertical line; bottom right). **(b)** As in (a) but for example sessions with optimal k > 4 (young, MA, and aged from left to right) selected during the sweep to set the maximum possible k (see Methods). In brief, as before, we selected the k that maximized average silhouette score across clusters among possible 2 ≤ k ≤ 8 over 10 repetitions of a cross-validation procedure (bottom left; SEM shaded). For each possible k, we compared 10-fold cross-validation train vs. test R^2 (top right) and the distribution of trial silhouette scores for each map vs. the average silhouette score (bottom right). In each example, at least one cluster had no trials with silhouette scores greater than or equal the average, indicating a low-quality cluster. Since these subpanels do not reflect selected models, the grid similarity matrix (top left) is unlabeled. **(c)** As in (a), for an example SM session fitted poorly by k > 2 (One Map = True). At the optimal k, the model's mean test R^2 for the real vs. shuffle firing rate tensors did not differ (one-sided Wilcoxon signed-rank test, $p > 0.05$). Since this session was not fit by k > 2, the grid network similarity matrix (top left) is unlabeled. **(d)** As in (c), for an example RF session not fit by k > 2. Here, a spatial cell network similarity matrix (top left) is shown unlabeled. **(e)** Relative density of optimal k values for RF sessions with > 10 spatial cells (left; n = 37 young & 39 aged) and for SM sessions with ≥ 10 grid cells (right; n = 46 young, 47 MA & 41 aged). The optimal k distribution did not differ significantly across age groups for either task (RF: 2-sample Kolmogorov-Smirnov test, A vs. Y: D = 0.023, p = 0.25, sign = -1, loc = 2.0; SM: A vs. Y: D = 0.13, p = 0.79, sign = -1, loc = 2.0; MA vs. Y: D = 0.077, p = 0.99, sign = 1, loc = 1.0; A vs. MA: D = 0.18, p = 0.37, sign = -1, loc = 2.0). Consistent with previous work,[23] most RF sessions were fit by k = 2 models. **(f)** RF spatial cell network trial-by-trial similarity matrices with spatial maps labeled by k = 2 - 4 models (left to right) from example sessions in each age group. The matrices omit dark and gain trials, and the right axis gives k-means map labels for each trial with arbitrary color order. Labeled matrices reveal similarity structure well captured by optimized k-means models. Subpanel titles indicate mouse, session number (S), and network spatial cell count. Color bar indicates trial-by-trial spatial correlation value. **(g)** As in Fig. 3a and (f), for SM grid network trial-by-trial similarity matrices, for which similarity structure is also well captured by optimized k-means models. VR context (top axis: context A [pink], context B [dark blue]) contrasts with k-means spatial map labels for each trial (right axis: map dominating context A trials [pink]; map dominating context B trials [dark blue], color of remaining maps, green or black arbitrarily). Each labeled map was also assigned a context identity (see Methods). Y27_S6, MA1_S6, and A19_S6 map labels contrast with map identities in Fig. 3a. Subpanel titles indicate mouse, session number (S), and network grid cell count. **(h)** As in Fig. 3b, remapping frequency of RF spatial cell networks (n = 37 young, 39 aged sessions). Remapping frequency did not differ between age groups in the RF task (young vs. aged, 0.0078 ± 0.0006 vs. 0.0122 ± 0.0018, Wilcoxon rank sum test, p = 0.40). **(i)** Alternation (alt.) performance (fraction [frac.] reward requested) vs. remapping frequency (left) or optimal k value (right) for all SM sessions across age groups (n = 47 young, 46 MA, and 41 aged sessions). Remapping frequency did not relate to alternation performance across all sessions (r = -0.14, p = 0.11) or among aged sessions (r = -0.15, p = 0.35). K value also did not relate to alternation performance across all sessions (r = -0.11, p = 0.22) or among aged sessions (r = -0.045, p = 0.78). Plotted as in the top of Fig. 2g, 2i, 2k, and 3d. Related to Fig. 3.

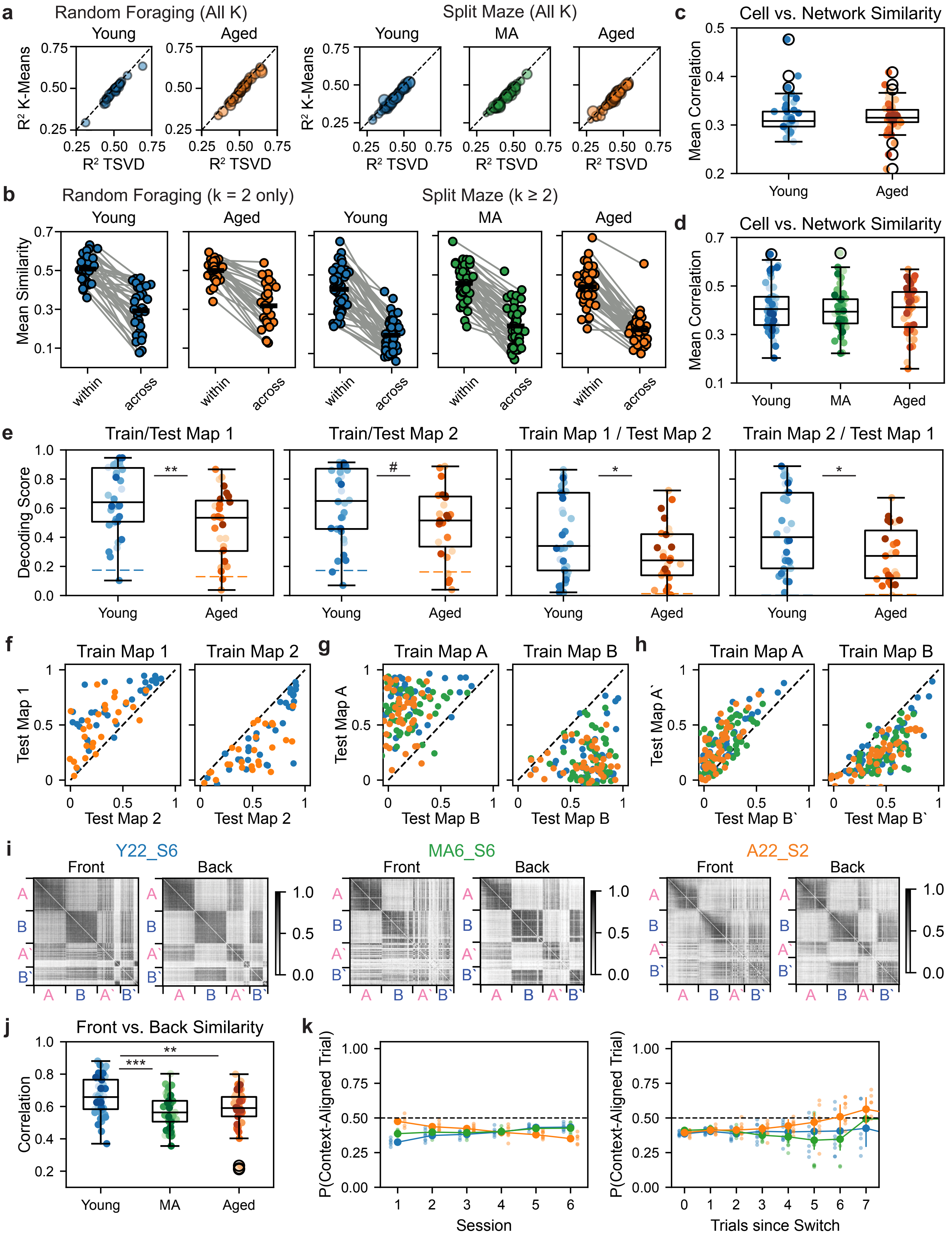
a
Random Foraging (All K)
Split Maze (All K)
Young
Aged
MA
R² K-Means
R² TSVD
c
Cell vs. Network Similarity
Mean Correlation
b
Random Foraging (k = 2 only)
Split Maze (k ≥ 2)
Mean Similarity
within
across
d
Cell vs. Network Similarity
e
Train/Test Map 1
Train/Test Map 2
Train Map 1 / Test Map 2
Train Map 2 / Test Map 1
Decoding Score
**
#
*
f
Train Map 1
Train Map 2
Test Map 1
Test Map 2
g
Train Map A
Train Map B
Test Map A
Test Map B
h
Test Map A`
Test Map B`
i
Y22_S6
MA6_S6
A22_S2
Front
Back
A
B
A`
B`
j
Front vs. Back Similarity
Correlation
***
k
P(Context-Aligned Trial)
Session
Trials since Switch

Supplementary Fig. 5: Remapping discreteness and coordination is preserved in aging, resulting in the conservation of positional information across maps and contexts.

(a) Rank-matched k-means vs. truncated singular value decomposition (tSVD) model performance (test R^2) for RF sessions (left; n = 39 young, 39 aged) and for SM sessions (right; n = 48 young, 51 MA, & 45 aged). Dot size scales with optimized k (k = 2 - 4). tSVD and k-means model performance related strongly for each age group across tasks (RF: Young, r = 0.98, p < 0.0001, Aged, r = 0.99, p < 0.0001; SM: Young, r = 0.98, p < 0.0001; MA, r = 0.98, p < 0.0001; Aged, r = 0.98, p < 0.0001). This indicates that k-means model assumptions of spatial maps comprising discrete trials are appropriate (see Methods). **(b)** Mean pairwise spatial similarity for trials within vs. across spatial maps for each RF session by age group where optimal k = 2 (left) and SM session where k ≥ 2 (right). Both RF age groups exhibited a significant mean similarity decrease (n = 34 young, 27 aged pairs; % mean similarity decrease, young, 42.85% ± 3.81%, Wilcoxon signed-rank test, young, p < 0.0001; aged, 37.35 ± 3.51%, p < 0.0001). There was no difference in the change in mean similarity across RF age groups (Wilcoxon rank sum test, p = 0.40). All SM groups also exhibited significant change in mean similarity (n = 47 young, 46 MA, and 41 aged pairs; % similarity decrease, young vs. MA vs. aged, 53.95 ± 2.38% vs. 48.23 ± 2.51% vs. 49.53 ± 2.14%). There was also no significant difference in the change in similarity across SM groups (Kruskal-Wallis test, H = 4.17, p = 0.12). Dots represent individual session data, and black bars indicate the mean across sets of plotted sessions. **(c)** Remapping coordination in RF sessions with at least three spatial cells (n = 42 young, 42 aged), computed as mean correlation between cell and network trial-by-trial similarity matrices (see Methods), plotted as in Supplementary Fig. 2b. Remapping coordination did not differ across age groups (young vs. aged, 0.318 ± 0.006 vs. 0.317 ± 0.005, Wilcoxon rank sum test, p = 0.26). **(d)** As in (c) for SM sessions with at least three grid cells, plotted as in Fig. 2d. Remapping coordination did not differ across age groups (n = 52 young, n = 57 MA, and n = 51 aged, young vs. MA vs. aged, 0.415 ± 0.014 vs. 0.4 ± 0.011 vs. 0.403 ± 0.013, Kruskal-Wallis test, H = 0.54, p = 0.76). **(e)** Decoder performance on spatial cell activity from all RF sessions with k = 2 across all possible training/testing map combinations (left to right) (n = 34 young, n = 27 aged), plotted as in Fig. 5h. Aged session decoder scores were lower than young ones for all train/test combinations (score, Map 1/1 young vs. aged, 0.6544 ± 0.0393 vs. 0.4813 ± 0.0446, Wilcoxon rank sum test, p = 0.0090; Map 2/2, 0.6264 ± 0.0437 vs. 0.4908 ± 0.0451, p = 0.0435; Map 1/2, 0.4151 ± 0.0482 vs. 0.2745 ± 0.0385, p = 0.0718; Map 2/1, 0.4382 ± 0.0488 vs. 0.2697 ± 0.0375, p = 0.0202). Dashed line indicates mean shuffle score for each map combination and age group. **(f)** Decoder performance trained and tested within maps in the RF track was greater than when trained and tested across maps (n = 68 young, 54 aged model pairs; young train/test same vs. different, Wilcoxon signed-rank test, p < 0.0001; aged train/test same vs. different, p < 0.0001). This indicates that spatial network positional information is discretized across RF spatial maps in both age groups. Dots indicate session values colored by age group. **(g)** As in (f), decoder score when trained and tested within vs. across contexts for the SM block phase. Decoder performance decreased when trained and tested across vs. within contexts for each age group (n = 96 young model pairs, 0.7076 ± 0.0197 vs. 0.164 ± 0.0243, Wilcoxon signed-rank test, p < 0.0001; n = 102 MA model pairs, 0.6623 ± 0.0168 vs. 0.1928 ± 0.0188, p < 0.0001; n = 90 aged model pairs, 0.6225 ± 0.0226 vs. 0.128 ± 0.0156, p < 0.0001). Within each age group, decoder score did not differ across contexts if train and test contexts matched (mean ± SEM train/test A vs. B score, n = 48 young, 0.7333 ± 0.0267 vs. 0.6818 ± 0.0287, Wilcoxon rank sum test, p = 0.15; n = 51 MA, 0.6429 ± 0.0255 vs. 0.6817 ± 0.0218, p = 0.27; n = 45 aged sessions, 0.6214 ± 0.0316 vs. 0.6238 ± 0.0325, p = 0.74). This indicates that grid network positional information is discretized across SM contexts for all age groups. **(h)** As in (g), decoder score when trained and tested across context-matched vs. -mismatched SM trial groups. Decoder performance was greater when trained and tested on context-matched (A x A`, B x B`) vs. -mismatched (A x B`, B x A`) trial groups for each age group (n = 96 young model pairs, 0.3842 ± 0.0256 vs. 0.2206 ± 0.0233, Wilcoxon signed-rank test, p < 0.0001; n = 102 MA model pairs, 0.4093 ± 0.0196 vs. 0.2234 ± 0.0158, p < 0.0001; n = 90 aged model pairs, 0.3529 ± 0.0230 vs. 0.175 ± 0.0160, p < 0.0001). Within each age group, decoder score was greater if trained and tested on B x B` than on A x A` contexts (mean ± SEM train/test A x A` vs. B x B` score, n = 48 young, 0.3054 ± 0.0369 vs. 0.4630 ± 0.0319, Wilcoxon rank sum test, p = 0.0021; n = 51 MA, 0.3269 ± 0.0266 vs. 0.4916 ± 0.0239, p < 0.0001; n = 45 aged sessions, 0.2834 ± 0.0284 vs. 0.4225 ± 0.0332, p = 0.0016). These results underscore that grid network positional information is discretized across SM contexts in different task phases for all age groups. **(i)** Example SM grid network similarity matrices generated from activity in the front (0 - 200 cm) vs. back (200 - 400 cm) of the VR track, omitting dark trials, including gain trials, and sorting alternation trials by context. In each case, the back track matrix exhibited greater context-specific structure compared to the

front track matrix. **(j)** Correlation of front vs. back track SM grid similarity matrices (n = 48 young, 51 MA, and 45 aged sessions), plotted as in Fig. 2d. This correlation differed across age groups (young vs. MA vs. aged, 0.66 ± 0.02 vs. 0.57 ± 0.01 vs. 0.58 ± 0.02, Kruskal-Wallis test, H = 17.0, p = 0.0002). Young sessions exhibited greater correlation compared to MA or aged sessions (post-hoc Conover test, young vs. MA, p = 0.00014, young vs. aged, p = 0.0046, MA vs. aged, p = 0.33). To account for this difference in the timing of context recognition during trials, all analyses in Fig. 3 were performed on back of track matrices. **(k)** As in Fig. 3d, for front track SM grid network activity on alternation trials (n = 7818 trials, pseudo R^2 = 0.1206, LLR p < 0.0001). Contrasting back track activity, the probability of map-context alignment during alternation was below chance for all age groups across sessions, and session predicted increasing map-context alignment probability only on young alternation trials (Young, Odds Ratio [OR] = 1.10, p = 0.00021; MA, p = 0.32; Aged, OR = 0.92, p = 0.0029). Trials since a context switch did not predict map-context alignment probability for front track grid network activity in any group (Young, p = 0.72, MA, p = 0.10, Aged, p = 0.32). Taken together with (j), this raises the possibility that the front track cue tower may weakly drive remapping in young mice in later sessions. However, in all age groups, the context cue tower in the front track alone was insufficient to produce above chance map-context alignment during alternation. Related to Fig. 3.

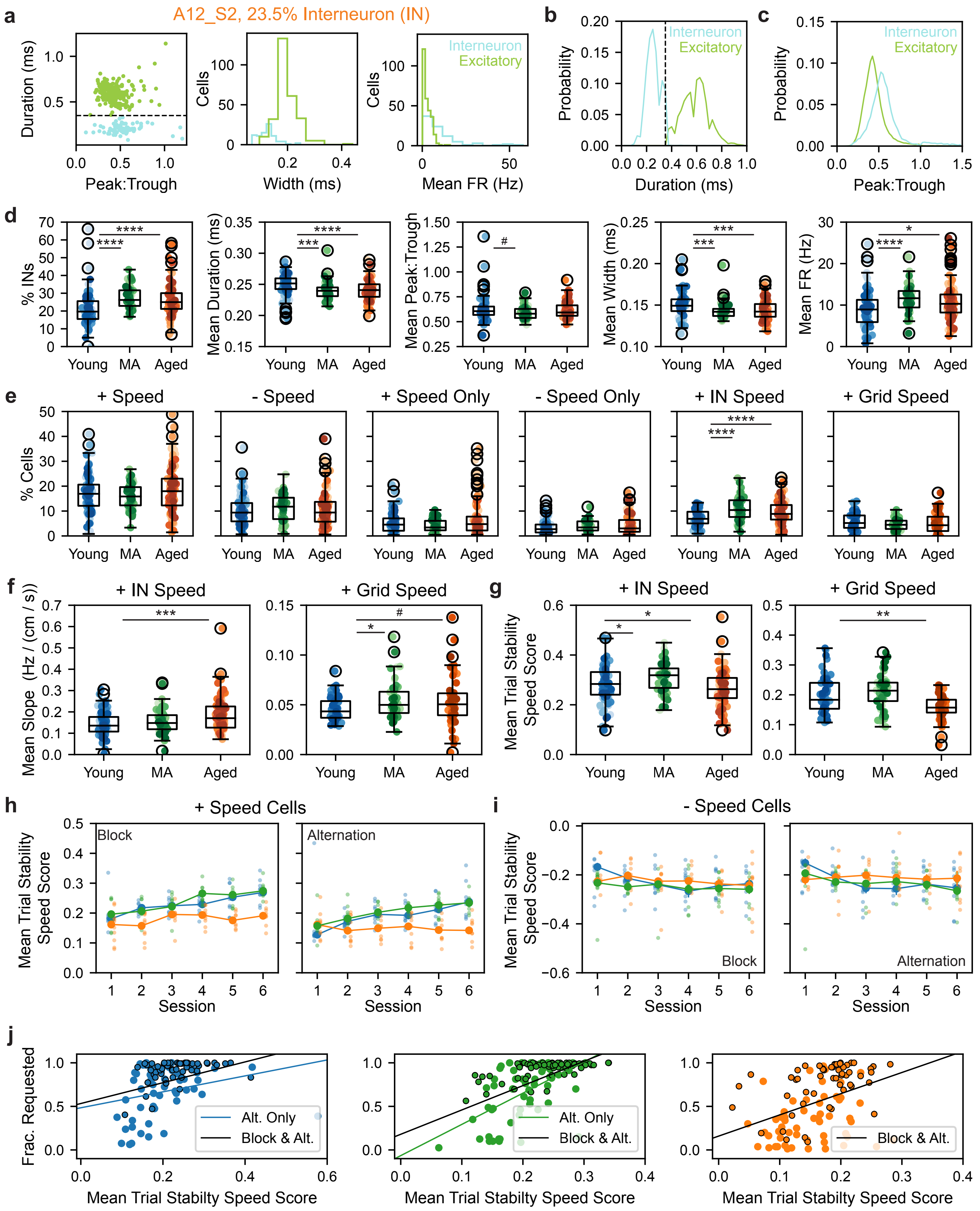

a
A12_S2, 23.5% Interneuron (IN)
Duration (ms)
Peak:Trough
Cells
Width (ms)
Mean FR (Hz)
Interneuron
Excitatory
b
Probability
Duration (ms)
c
Peak:Trough
d
% INs
Mean Duration (ms)
Mean Peak:Trough
Mean Width (ms)
Mean FR (Hz)
Young
MA
Aged
e
+ Speed
- Speed
+ Speed Only
- Speed Only
+ IN Speed
+ Grid Speed
% Cells
f
Mean Slope (Hz / (cm / s))
g
Mean Trial Stability Speed Score
h
+ Speed Cells
Block
Alternation
Session
i
- Speed Cells
j
Frac. Requested
Alt. Only
Block & Alt.
Mean Trial Stabilty Speed Score

Supplementary Fig. 6. Increased speed gain & speed tuning instability also occur in aged mouse fast-spiking interneurons, conjunctive grid-speed cells, & speed-only cells.

(a) Classification of putative inhibitory interneurons (INs) (light blue) vs. excitatory (EX) cells (lime green) from an example aged session (see Methods). Putative INs vs. excitatory cells clustered in waveform duration and peak:trough ratio space and were well-separated by a waveform duration threshold (left) (dashed black line). Histograms of session IN vs. excitatory cells by waveform width (ms) (middle) and mean firing rate (FR [Hz]) (right) confirmed that INs exhibited narrower waveforms and higher FR. Colors for INs vs EX cells maintained throughout. **(b)** Probability density of the waveform duration of classified EX cells vs. INs (light blue) (n = 49,193 EX cells and 15,123 INs). Waveform duration EX cells exceeded that of INs (EX vs. INs, 0.5807 ± 0.0005 vs. 0.2432 ± 0.0005, Wilcoxon rank sum test, $p < 0.0001$). Dashed black line indicates the IN waveform duration threshold. A small minority of INs exceeded the duration threshold, since they were classified by the secondary FR threshold (> 40 Hz) (see Methods). Bin size was 0.025 ms. **(c)** As in (b) for waveform peak:trough ratio (PTR), which also differed significantly across cell types (EX vs. INs, 0.4445 ± 0.0010 vs. 0.6245 ± 0.0034, $p < 0.0001$). Bin size was 0.025. **(d)** Differences in IN density (first; n = 98 young, 58 MA, & 97 aged sessions) and waveform and FR properties (n = 97 young, 58 MA, 97 aged sessions with INs) across age groups, including mean session waveform duration (second), PTR (middle), mean waveform halfwidth (fourth), and mean FR (last). IN density differed across age groups (% INs, young vs. MA vs. aged, 21.22 ± 0.92 vs. 27.35 ± 0.81 vs. 26.24 ± 0.87, Kruskal-Wallis test, H = 39.21, $p < 0.0001$; post-hoc Conover test, young vs. MA, $p < 0.0001$; young vs. aged, $p < 0.0001$). As did IN waveform duration (0.2481 ± 0.0018 vs. 0.2406 ± 0.0019 vs. 0.2402 ± 0.0016, H = 22.59, $p < 0.0001$; young vs. MA, p = 0.00014; young vs. aged, p = 0.000063). IN waveform PTR did not differ with age (0.6281 ± 0.0121 vs. 0.5905 ± 0.0083 vs. 0.6166 ± 0.0079, H = 5.83, p = 0.054; young vs. MA, p = 0.059; young vs. aged, p = 0.66). IN waveform halfwidth differed significantly with aging (0.1503 ± 0.0015 vs. 0.1431 ± 0.0013 vs. 0.1433 ± 0.0012, H = 21.5, $p < 0.0001$; young vs. MA, p = 0.00015, young vs. aged, p = 0.00014). Finally, IN mean FR differed significantly across age groups (9.0683 ± 0.4352 vs. 11.4235 ± 0.424 vs. 10.7849 ± 0.4427, H = 18.58, $p < 0.0001$; young vs. MA, p = 0.000062, young vs. aged, p = 0.0107). These results collectively indicate possible differences in the relative density of MEC IN subtypes or changes in MEC IN function with age. Plotted as in Figs. 4c and 6d-e. **(e)** Density of various speed-tuned cell populations: + and - speed cells (first, second), + and - non-spatial speed cells (third, fourth), speed-tuned interneurons (fifth); and speed-tuned grid cells (last) by age group (all except for last: n = 98 young, 58 MA, & 97 aged sessions; last: n = 54 young, 58 MA, 55 aged sessions). + and - speed cell density did not differ with age (% cells + speed cells, young vs. MA vs. aged, 16.67 ± 0.77 vs. 16.13 ± 0.64 vs. 18.41 ± 0.95, Kruskal-Wallis test, H = 1.71, p = 0.42; post-hoc Conover test, young vs. MA, p = 0.82; young vs. aged, p = 0.76; % cells - speed cells, 10.11 ± 0.6 vs. 11.02 ± 0.7 vs. 10.52 ± 0.74, H = 2.21, p = 0.33; young vs. MA, p = 0.54; young vs. aged, p = 0.99). Neither did the density of + and - non-spatial speed cells (% + speed only cells, 4.63 ± 0.41 vs. 3.96 ± 0.35 vs. 6.88 ± 0.76, H = 4.00, p = 0.14; young vs. MA, p = 0.84; young vs. aged, p = 0.26; % - speed only cells, 3.24 ± 0.3 vs. 3.59 ± 0.34 vs. 4.18 ± 0.39, H = 2.99, p = 0.37; young vs. MA, p = 0.82; young vs. aged, p = 0.35). + speed-tuned IN density increased starting in middle age (% + speed INs, 6.92 ± 0.35 vs. 10.97 ± 0.63 vs. 9.39 ± 0.48, H = 29.1, $p < 0.0001$; young vs. MA, $p < 0.0001$; young vs. aged, p = 0.0002). Finally, + speed-tuned grid cell density was uniform across age groups (% + speed grid cells, 5.64 ± 0.46 vs. 4.64 ± 0.3 vs. 5.34 ± 0.57, H = 2.13, p = 0.35; young vs. MA, p = 0.52 young vs. aged, p = 0.53). These results suggest that differences in speed cell density are unlikely to account for altered speed tuning with age (see Figs. 6d-e). Plotted as in Figs. 4c and 6d-e. **(f)** As in Fig. 6d, mean FR-speed slope, or speed gain, for + speed-tuned INs (left, n = 94 young, 58 MA, 95 aged sessions) and + speed-tuned grid cells (right, n = 52 young, 57 MA, 50 aged sessions). Speed gain was increased among + speed INs and + speed grid cells in aged vs. young sessions (+ speed INs: 0.1458 ± 0.0057 vs. 0.1588 ± 0.0083 vs. 0.1857 ± 0.0082, H = 13.24, p = 0.0013; young vs. MA, p = 0.2307, young vs. aged, p = 0.0008; + speed grid cells: 0.0460 ± 0.0016 vs. 0.0540 ± 0.0024 vs. 0.0536 ± 0.0036, H = 6.67, p = 0.0356; young vs. MA, p = 0.0441, young vs. aged, p = 0.0911). **(g)** As in Fig. 6e and (f) for mean trial stability speed score. + speed INs speed tuning stability decreased in aged vs. young sessions (0.2813 ± 0.0072 vs. 0.3105 ± 0.0076 vs. 0.2623 ± 0.0075, H = 19.48, $p < 0.0001$; young vs. MA, p = 0.0156, young vs. aged, p = 0.0316). Similarly, + speed grid cells tuning stability decreased in aged vs. young sessions (0.1991 ± 0.0087 vs. 0.2086 ± 0.0075 vs. 0.1581 ± 0.0059, H = 22.09, $p < 0.0001$; young vs. MA, p = 0.1591, young vs. aged, p = 0.0014). **(h)** As in Fig. 2f for SM block (left) and alternation (right) trial stability speed score (n = 3933 young, 4062 MA, and 3423 aged + speed cell cells). Session predicted improved block speed coding stability among young but not aged mice (Session x Young, $\beta = 0.016$, $p < 0.0001$;

Session x Aged, β = 0.003, p = 0.19) (Intercept = 0.169 ± 0.011, p < 0.0001). Similarly, session predicted improvement in young but not aged alternation speed coding stability (Session x Young, β = 0.019, p < 0.0001; Session x Aged, β = -0.004, p = 0.263) (Intercept = 0.111 ± 0.012, p < 0.0001). This suggests that + speed cell speed coding stability fails to improve over sessions among aged mice. **(i)** As in (h) for SM - speed cells (n = 1472 young, 2071 MA, and 1415 aged – speed cells). Session predicted a modest improvement in block speed coding stability in young and aged mice (Session x Young, β = -0.010, p < 0.0001; Session x Aged, β = -0.009, p = 0.011) (Intercept = -0.180 ± 0.017, p < 0.0001). Conversely, session predicted a modest improvement in young but not aged alternation speed coding stability (Session x Young, β = -0.015, p < 0.0001; Session x Aged, β = -0.004, p = 0.263) (Intercept = -0.166 ± 0.018, p < 0.0001). This suggested that - speed cell speed coding stability fails to improve over sessions among aged mice during alternation. These models of – speed cell tuning stability converged only when using maximum likelihood estimators and excluding animal cohort as a fixed effect. **(j)** As in Fig. 2h, for SM task performance vs. mean trial stability speed score of + speed cells (n = 108 young, 116 MA, 108 aged task phases). Colored lines indicate regression fits for alternation alone. Trial stability speed score related to task performance across phases (Young: r = 0.35, p = 0.0002, MA: r = 0.56, p < 0.0001, Aged: r = 0.40, p < 0.0001) and when considering alternation alone (Young: r = 0.27, p = 0.0497, MA: r = 0.55, p < 0.0001, Aged: r = 0.24, p = 0.097). Related to Fig. 6.

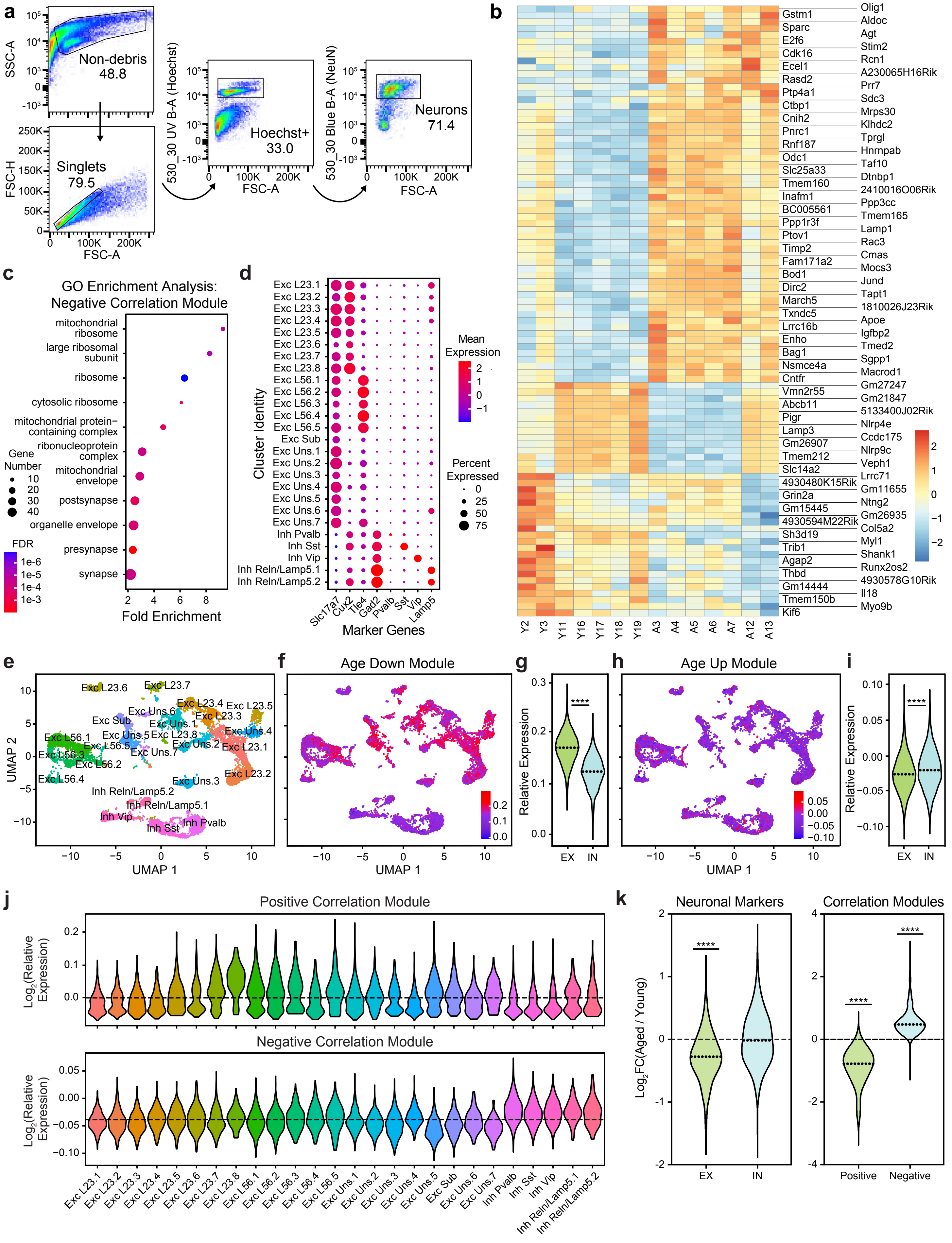
a
Non-debris
48.8
Singlets
79.5
Hoechst+
33.0
Neurons
71.4
SSC-A
FSC-H
FSC-A
530_30 UV B-A (Hoechst)
530_30 Blue B-A (NeuN)
b
c
GO Enrichment Analysis:
Negative Correlation Module
mitochondrial ribosome
large ribosomal subunit
ribosome
cytosolic ribosome
mitochondrial protein–containing complex
ribonucleoprotein complex
mitochondrial envelope
postsynapse
organelle envelope
presynapse
synapse
Gene Number
FDR
Fold Enrichment
d
Cluster Identity
Marker Genes
Mean Expression
Percent Expressed
e
UMAP 1
UMAP 2
f
Age Down Module
g
Relative Expression
EX
IN
h
Age Up Module
i
j
Positive Correlation Module
Negative Correlation Module
Log2(Relative Expression)
k
Neuronal Markers
Correlation Modules
Log2FC(Aged / Young)
Positive
Negative

Supplementary Fig. 7. Cell type- and layer-specificity of transcriptomic changes in the aging MEC

(a) Fluorescence-activated cell sorting (FACS) gating strategy to isolate neuronal nuclei dissociated from MEC (see Methods). Briefly, nuclei were gated by forward (FSC-A) and side (SSC-A) scatter to remove debris (left top) and by height (FSC-H) and size (FSC-A) to remove doublets (left bottom). Hoechst+ and NeuN+ nuclei were then isolated iteratively (middle, right). Subplots are labeled with the type and percent of nuclei isolated at each sorting step. **(b)** Heatmap of the top 100 most differentially expressed genes as determined by adjusted p-value with a log2 absolute fold change > 1 (n = 7 young, 7 aged mice; Wald test followed by Benjamini-Hochberg multiple hypothesis correction). Color bar indicates Z-score of expression level with age (red = increased with age; blue = decreased with age). **(c)** As in Fig. 7h, the cellular components enriched among negative coherence-correlated module genes (Fisher's Exact test followed by FDR calculation) via GO Enrichment analysis. **(d)** Dot plot of top representative cell marker genes used to distinguish clusters in snRNA-seq data. **(e)** UMAP plot of neurons from MEC snRNA-seq with neuronal subtype annotations layered on or above their respective clusters. If known, excitatory cell (Exc.) clusters are labeled with their gross cortical layer (e.g. 23 for II/III or 56 for V/VI) and numbered arbitrarily within the layer group. Excitatory cell clusters of unknown layer (Exc Uns) are numbered arbitrarily. Inhibitory IN clusters (Inh) are labeled with their primary cellular marker gene (e.g. *Pvalb*, *Sst*, *Vip*). A cluster corresponding to subiculum excitatory cells was also identified (Exc Sub). **(f)** UMAP plot with the relative expression of the gene module that decreased expression during aging (Age Down) (n = 242 genes represented in snRNA-seq data). Color bar indicates expression relative to control feature sets. Y-axis is shared with (e). **(g)** Violin plot of the relative expression of the age decreased gene module between INs and excitatory (EX) neurons. Age-decreased genes are enriched among excitatory cells (EX) (0.1682 ± 0.0003 vs. 0.1215 ± 0.0006, EX vs. IN, Wilcoxon rank sum test, $p < 0.0001$). Dotted lines indicate median relative expression. **(h)** As in (f), for the gene module that increased expression during aging (Age Up) (n = 160 genes). **(i)** As in (g), for the age increased gene module. Age-increased genes enriched among INs (-0.0233 ± 0.0003 vs. -0.0179 ± 0.0006, EX vs. IN, Wilcoxon rank sum test, $p < 0.0001$). **(j)** Violin plots of positive (top) & negative (bottom) coherence correlated core gene module relative expression across identified neuronal clusters. Expression of the positive correlation module is enriched in some Layer II/III and V/VI excitatory cell clusters while expression of the negative correlation module is enriched among IN clusters. Dotted lines indicate median relative expression. Colors correspond to labeled clusters in (e). **(k)** Violin plots of excitatory neuron (EX) (n = 323 genes) and IN (n = 199 genes) marker gene expression change with age (top) (Wilcoxon signed-rank test, $p < 0.0001$) and positive and negative coherence core gene modules expression change with age (bottom) in bulk expression data. EX cell marker expression changed with age (-0.2808 ± 0.02134, Wilcoxon signed-rank test, $p < 0.0001$), but IN marker expression did not (0.0008763 ± 0.02899, Wilcoxon signed-rank test, $p = 0.80$). Positive correlation gene module expression decreased with age (-0.8599 ± 0.1037, Wilcoxon signed-rank test, $p < 0.0001$), and negative correlation gene module expression increased with age (0.5678 ± 0.02597, Wilcoxon signed-rank test, $p < 0.0001$). Cross-variable comparisons within or across subpanels should not be made given different gene set sizes and expression levels. Dashed lines indicate equivalent expression across ages. Dotted lines indicate medians. Data are represented as the log2 fold change (FC) of aged over young expression. Lower opacity emphasizes that these data reflect bulk RNA-seq gene expression. Related to Fig. 7.

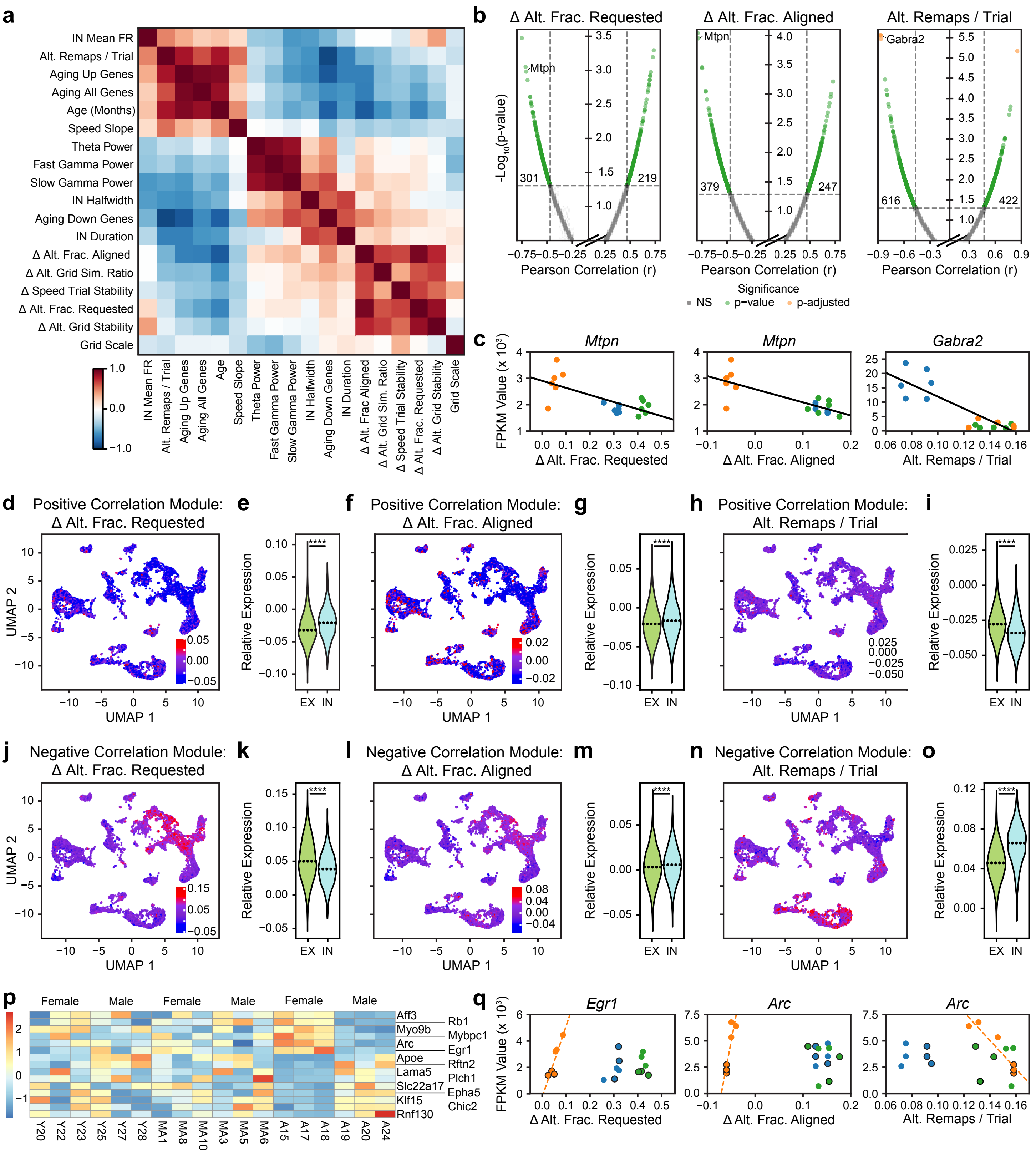
a
IN Mean FR
Alt. Remaps / Trial
Aging Up Genes
Aging All Genes
Age (Months)
Speed Slope
Theta Power
Fast Gamma Power
Slow Gamma Power
IN Halfwidth
Aging Down Genes
IN Duration
Δ Alt. Frac. Aligned
Δ Alt. Grid Sim. Ratio
Δ Speed Trial Stability
Δ Alt. Frac. Requested
Δ Alt. Grid Stability
Grid Scale
1.0
0.0
−1.0
b
Δ Alt. Frac. Requested
Δ Alt. Frac. Aligned
Alt. Remaps / Trial
-Log10(p-value)
Mtpn
Gabra2
301
219
379
247
616
422
Pearson Correlation (r)
Significance
NS
p−value
p-adjusted
c
Mtpn
Mtpn
Gabra2
FPKM Value (x 10^3)
Δ Alt. Frac. Requested
Δ Alt. Frac. Aligned
Alt. Remaps / Trial
d
Positive Correlation Module: Δ Alt. Frac. Requested
e
f
Positive Correlation Module: Δ Alt. Frac. Aligned
g
h
Positive Correlation Module: Alt. Remaps / Trial
i
UMAP 2
UMAP 1
Relative Expression
****
EX
IN
j
Negative Correlation Module: Δ Alt. Frac. Requested
k
l
Negative Correlation Module: Δ Alt. Frac. Aligned
m
n
Negative Correlation Module: Alt. Remaps / Trial
o
p
Female
Male
Female
Male
Female
Male
Aff3
Rb1
Myo9b
Mybpc1
Arc
Egr1
Apoe
Rftn2
Lama5
Plch1
Slc22a17
Epha5
Klf15
Chic2
Rnf130
Y20
Y22
Y23
Y25
Y27
Y28
MA1
MA8
MA10
MA3
MA5
MA6
A15
A17
A18
A19
A20
A24
q
Egr1
Arc
Arc
FPKM Value (x 10^3)
Δ Alt. Frac. Requested
Δ Alt. Frac. Aligned
Alt. Remaps / Trial

Supplementary Fig. 8. Transcriptomic correlates of spatial cognitive inflexibility in the aged MEC

(a) As in Fig. 7e, correlation among LMM-fitted animal-mean of neural parameters computed for SM sessions across cell types (INs, speed cells, and grid cells), animal age in months, and aging DEG expression (total, up-regulated with age [Up], and down-regulated with age [Down]) (see Supplementary Data 3). For behavioral and neural metrics that changed significantly over sessions, we computed the change (Δ) in the LMM-fitted animal means of the last three sessions minus that of the first three sessions (see Methods) as a measure of spatial cognitive flexibility. Across age groups, the two strongest neural correlates of aging genes were features unique to the SM (compared to RF) task: alternation remapping frequency (alt. remaps / trial) ($r = 0.65$, $p = 0.0036$) and change in the fraction of trials with context-aligned grid network activity during alternation (Δ alt. frac. aligned) ($r = -0.69$, $p = 0.0017$). Change in the fraction of alternation rewards requested over sessions (Δ alt. frac. requested) also related to the expression of aging DEGs ($r = -0.52$, $p = 0.0268$). **(b)** As in Fig. 7f, volcano plots of the correlation of gene expression with Δ alt. frac. requested (left), Δ alt. frac. aligned (middle), and alt. remaps / trial (right) across the SM dataset. Numbers denote the total of negatively (left) and positively (night) metric-correlated genes (linear regression, $p < 0.05$ [green]). Orange dots denote transcriptomic correlates that retained significance after a Benjamini-Hochberg correction procedure (false discovery rate [FDR] = 0.10). **(c)** As in Fig. 7g, example genes that are correlated ($p < 0.05$) with Δ alt. frac. requested (left, *Mtpn,* $r = -0.71$, $p = 0.0010$), Δ alt. frac. aligned (middle, *Mtpn,* $r = -0.79$, $p = 0.0001$), and alt. remaps / trial (right, *Gabra2*, $r = -0.87$, $p < 0.0001$) across SM age groups (n = 6 young, 6 MA, 6 aged mice). Notably, the expression of 369 genes, including *Mtpn,* was jointly correlated with the improvement of behavioral flexibility and grid network context-alignment over experience, while genes also related to remapping frequency were less overlapping (33 shared correlates). Dots are colored by age group. **(d)** UMAP plot with the relative expression of the gene core module positively correlated with the change in SM alternation behavior over sessions (Δ alt. frac. requested) (n = 211 genes). **(e)** Violin plot of the relative expression of the gene core module positively correlated with Δ alt. frac. requested across MEC excitatory cells (EX) and inhibitory interneurons (IN) (EX vs. IN, -0.03030 ± 0.0002382 vs. -0.02065 ± 0.0004755, Wilcoxon rank sums test, $p < 0.0001$) (see Fig. 7i for UMAP subdivision into EX vs IN groups and Supplementary Figs. 7d-e for full UMAP cluster annotation). This indicates that decreased expression of this module, especially among MEC INs, relates to reduced alternation behavior improvement over sessions in aging (see Fig. 1g). Dotted lines indicate median relative expression. **(f)** UMAP plot with the relative expression of the gene core module positively correlated with the change in SM alternation grid network context alignment over sessions (Δ alt. frac. aligned) (n = 232 genes). Y-axis is shared with (d). **(g)** Violin plot of the relative expression of the gene core module positively correlated with Δ alt. frac. aligned across MEC cell types (EX vs. IN, -0.01985 ± 0.0002430 vs. -0.01539 ± 0.0004880, Wilcoxon rank sums test, $p < 0.0001$). This suggests that decreased expression of this module, especially among MEC INs, relates to reduced improvement in grid network context alignment during alternation in aging (see Fig. 3h). Dotted lines indicate median relative expression. **(h)** UMAP plot with the relative expression of the gene core module positively correlated with SM alternation remapping frequency (alt. remaps / trial) (n = 349 genes). Y-axis is shared with (d). **(i)** Violin plot of the relative expression of the gene core module positively correlated with alt. remaps / trial across MEC cell types (EX vs. IN, -0.02746 ± 0.0001225 vs. -0.03328 ± 0.0002489, Wilcoxon rank sums test, $p < 0.0001$). Increased expression of this module, especially by MEC EX cells, relates to increased aged remapping frequency. Dotted lines indicate median relative expression. **(j)** As in (d), for the gene core module negatively correlated with Δ alt. frac. requested (n = 285 genes). **(k)** As in (e), for the gene core module negatively correlated with Δ alt. frac. requested (EX vs. IN, 0.05143 ± 0.0003125 vs. -0.03837 ± 0.0004838, Wilcoxon rank sums test, $p < 0.0001$). Notably, this module appeared especially enriched among Layer II/III EX cells, suggesting that increased expression of this module by MEC Layer II/III EX cells relates to diminished SM alternation behavior improvement in aging. **(l)** As in (f), for the gene core module negatively correlated with Δ alt. frac. aligned (n = 354 genes). Y-axis is shared with (j). **(m)** As in (g), for the gene core module negatively correlated with Δ alt. frac. aligned (EX vs. IN, 0.003371 ± 0.0002069 vs. 0.006028 ± 0.0003797, Wilcoxon rank sums test, $p < 0.0001$). This module also appeared enriched among Layer II/III EX cells, consistent with increased module expression by those cells relating to diminished improvement in grid network context alignment during alternation in aging. **(n)** As in (h), for the gene core module negatively correlated with alt. remaps/trial (n = 586 genes). Y-axis is shared with (j). **(o)** As in (i), for the gene core module negatively correlated with alt. remaps/trial (EX vs. IN, 0.04619 ± 0.0001863 vs. 0.06487 ± 0.0003912, Wilcoxon rank sums test, $p < 0.0001$). The enrichment of this module among INs suggests that decreased module expression by INs relates to increasing remapping frequency observed with aging (see Fig. 3b). **(p)** Plotted

as in Supplementary Fig. 7b, heatmap of the 15 autosomal genes differentially expressed across sexes among aged SM mice (n = 3 female, 3 male; Wald test followed by Benjamini-Hochberg multiple hypothesis correction). Notably, immediate early genes *Arc* and *Egr1* are down-regulated in aged male vs. female SM mice. **(q)** As in (c), example aged sex DEGs with expression correlated ($p < 0.05$) with Δ alt. frac. requested (left, *Egr1*, $r = 0.91$, $p = 0.0127$), Δ alt. frac. aligned (middle, *Arc*, $r = 0.89$, $p = 0.0165$), and alt. remaps / trial (right, *Arc*, $r = -0.94$, $p = 0.0052$) among aged SM mice. *Arc* and *Egr1* expression was also related among aged mice (not shown, $r = 0.82$, $p = 0.0430$). Dots are colored by age group and outlined if corresponding to male mice. Dashed lines denote linear regression fits among aged mice. Decreased IEG expression in aged males vs. females related to reduced behavioral and grid network context alignment improvement over SM task experience and greater remapping frequency. Related to Fig. 7.
