# Supplementary material for: Spatial coding dysfunction and network instability in the aging medial entorhinal cortex
Source: Nat Commun. 2025 Oct 3;16:8770. doi: 10.1038/s41467-025-63229-0 (PMC12494969; doi:10.1038/s41467-025-63229-0)
Supplement: Supplementary file 2 — Description of Additional Supplementary File [file 41467_2025_63229_MOESM2_ESM.docx]

Description of Additional Supplementary Files

Supplementary Data 1: Complete linear mixed effects modeling results.

Excel file containing additional data too large to fit in a PDF, related to Figs. 1, 2, 6 and Supplementary Figs. 1, 2, 3 and 6.

Supplementary Data 2: MEC Differential Gene Expression in Random Foraging Mice.

Excel file containing additional data too large to fit in a PDF, related to Fig. 7.

Supplementary Data 3: MEC Differential Gene Expression in Split Maze Mice.

Excel file containing additional data too large to fit in a PDF, related to Supplementary Fig. 8.
