# Supplementary material for: Spatial coding dysfunction and network instability in the aging medial entorhinal cortex
Source: Nat Commun. 2025 Oct 3;16:8770. doi: 10.1038/s41467-025-63229-0 (PMC12494969; doi:10.1038/s41467-025-63229-0)
Supplement: Supplementary file 6 — Reporting Summary [file 41467_2025_63229_MOESM6_ESM.pdf]

nature portfolio

Corresponding author(s): Lisa M. Giocomo

Last updated by author(s): 07/09/2025

Reporting Summary

Nature Portfolio wishes to improve the reproducibility of the work that we publish. This form provides structure for consistency and transparency in reporting. For further information on Nature Portfolio policies, see our Editorial Policies and the Editorial Policy Checklist.

Statistics

For all statistical analyses, confirm that the following items are present in the figure legend, table legend, main text, or Methods section.

n/a	Confirmed	
☐	☒	The exact sample size (n) for each experimental group/condition, given as a discrete number and unit of measurement
☐	☒	A statement on whether measurements were taken from distinct samples or whether the same sample was measured repeatedly
☐	☒	The statistical test(s) used AND whether they are one- or two-sided<br>*Only common tests should be described solely by name; describe more complex techniques in the Methods section.*
☐	☒	A description of all covariates tested
☐	☒	A description of any assumptions or corrections, such as tests of normality and adjustment for multiple comparisons
☐	☒	A full description of the statistical parameters including central tendency (e.g. means) or other basic estimates (e.g. regression coefficient) AND variation (e.g. standard deviation) or associated estimates of uncertainty (e.g. confidence intervals)
☐	☒	For null hypothesis testing, the test statistic (e.g. F, t, r) with confidence intervals, effect sizes, degrees of freedom and P value noted<br>*Give P values as exact values whenever suitable.*
☒	☐	For Bayesian analysis, information on the choice of priors and Markov chain Monte Carlo settings
☒	☐	For hierarchical and complex designs, identification of the appropriate level for tests and full reporting of outcomes
☐	☒	Estimates of effect sizes (e.g. Cohen's d, Pearson's r), indicating how they were calculated

Our web collection on statistics for biologists contains articles on many of the points above.

Software and code

Policy information about availability of computer code

Data collection	Electrophysiological data were collected using Phase 3B Neuropixels 1.0 silicon probes, digitized with a CMOS amplifier and multiplexer built into the electrode array, and then written to disk using SpikeGLX software.
Data analysis	Analyses were carried out using MATLAB, Python, and R scripts. Python and R were used to perform statistical calculations. Cell clusters were isolated using Kilosort 2.5 and reviewed using Phy 2.0. All custom analysis code has been uploaded to GitHub and is public: https://doi.org/10.5281/zenodo.15851471.

For manuscripts utilizing custom algorithms or software that are central to the research but not yet described in published literature, software must be made available to editors and reviewers. We strongly encourage code deposition in a community repository (e.g. GitHub). See the Nature Portfolio guidelines for submitting code & software for further information.

Data

Policy information about availability of data

All manuscripts must include a data availability statement. This statement should provide the following information, where applicable:

- Accession codes, unique identifiers, or web links for publicly available datasets
- A description of any restrictions on data availability
- For clinical datasets or third party data, please ensure that the statement adheres to our policy

Pre-processed behavioral data, neural data, and immunohistochemistry images associated with this manuscript are available via Dryad (https://doi.org/10.5061/

dryad.8cz8w9h0d). Processed neural data included spike times and myriad waveform features for all recorded cells from all sessions and mice, facilitating replication of manuscript findings and many additional analyses. Raw neural data may be requested by contacting the corresponding author, given their large size, the significant time burden of data processing, and the limited number of additional analyses raw vs. processed neural data enable. Transcriptomic bulk and single nucleus RNA sequencing data from this study are separately available via the NCBI GEO database (Dataset 1 [Fig. 7 and Supplementary Fig. 7]: Accession Number: GSE263347; Dataset 2 [Supplementary Fig. 8]: Accession Number: GSE281777). Supplementary tables containing the results of transcriptomic data analyses of each dataset are included with this publication. Most transcriptomic data analyses (Fig. 7 and Supplementary Figs. 7 and 8) used commonly available R and Python packages that are specified in the manuscript Methods. Source data are also provided here.

Research involving human participants, their data, or biological material

Policy information about studies with human participants or human data. See also policy information about sex, gender (identity/presentation), and sexual orientation and race, ethnicity and racism.

Reporting on sex and gender	NA
Reporting on race, ethnicity, or other socially relevant groupings	NA
Population characteristics	NA
Recruitment	NA
Ethics oversight	NA

Note that full information on the approval of the study protocol must also be provided in the manuscript.

Field-specific reporting

Please select the one below that is the best fit for your research. If you are not sure, read the appropriate sections before making your selection.

☒ Life sciences ☐ Behavioural & social sciences ☐ Ecological, evolutionary & environmental sciences

For a reference copy of the document with all sections, see nature.com/documents/nr-reporting-summary-flat.pdf

Life sciences study design

All studies must disclose on these points even when the disclosure is negative.

Sample size	Samples sizes were based on convention in the field. The sample sizes were not calculated by power analyses. However, our sample sizes are comparable or higher than those previously used in the literature (Low et al., 2021; Campbell, Attinger, et al. 2021; Masuda et al., 2023; Wen and Sorscher et al. 2024).
Data exclusions	Cells with low firing rates (<350 spikes) and recording sessions with <10 cells were excluded. Mice were deemed fully trained on the virtual reality tasks and ready to record when they completed 250 trials within one hour for two consecutive days. Mice that never learned the task or who displayed signs of illness, distress, or sudden behavioral abnormalities (i.e. not running) during recording were excluded from further experiments. If a set of recordings was terminated early for such a reason, the last two sessions of behavioral data were excluded.
Replication	Up to 10 mice were used as a cohort for each batch of experiments. Key experiments were repeated with at least two different cohorts of mice, and the results were reproducible.
Randomization	Animals were assigned into groups based on age, not randomization. Covariates such as cohort, behavioral task, and sex were controlled for using linear mixed effects models with these covariates as fixed effects.
Blinding	Experimenters were not blinded to experimental groups for neural and behavioral data collection and analysis. Experimental groups were based on animal age, which is physically obvious based on animal size and physical characteristics. It would be impossible to blind the experimenter to the experimental conditions while still being physically present during the experiment. Presence during the experiment is required by the ethics committee overseeing the experiment. Immunohistochemical image analysis was semi-automated and performed by an experimenter blind to the age status of images.

Reporting for specific materials, systems and methods

We require information from authors about some types of materials, experimental systems and methods used in many studies. Here, indicate whether each material, system or method listed is relevant to your study. If you are not sure if a list item applies to your research, read the appropriate section before selecting a response.

Materials & experimental systems

n/a	Involved in the study	
☐	☒	Antibodies
☒	☐	Eukaryotic cell lines
☒	☐	Palaeontology and archaeology
☐	☒	Animals and other organisms
☒	☐	Clinical data
☒	☐	Dual use research of concern
☒	☐	Plants

Methods

n/a	Involved in the study	
☒	☐	ChIP-seq
☐	☒	Flow cytometry
☒	☐	MRI-based neuroimaging

Antibodies

Antibodies used	For FACS, we used anti-NeuN-AlexaFluor488 antibody (Millipore, Cat. MAB377X, RRID: AB_2149209) to label neurons. For IHC, we used rabbit anti-PV antibody [Swant, RRID: AB_10000344]; donkey anti-rabbit AlexaFluor 555 [Life Technologies, Cat# A-31572, RRID: AB_162543]; fluorescein conjugated Wisteria floribunda agglutinin (WFA) [4ug/mL, Vector Labs, Cat# FL-1351-2]; and Hoechst 33342 [2 ug/mL, Life Technologies, Cat# H3570]
Validation	Validation of each of the FACS and IHC antibodies was performed by the manufacturer. The FACS antibody, PV primary antibody, AlexaFluor secondary antibody, WFA stain, and Hoechst stain have also been referenced by more than 30 peer-reviewed publication each.

Animals and other research organisms

Policy information about studies involving animals; ARRIVE guidelines recommended for reporting animal research, and Sex and Gender in Research

Laboratory animals	Mice were C57Bl/6 and aged 2-4 months, 12-13 months, and 21-24 months at the time of recording and tissue collection, obtained from Charles River and Jackson as specified in the Methods. Animals were housed at 21-23°C and 30-40% humidity on a reverse light-dark 12 hour cycle.
Wild animals	No wild animals were used in this study.
Reporting on sex	Male and female were used as data subjects to the electrophysiology experiments and RNA sequencing experiments. Sex is included as a fixed effect in all linear mixed effects model. Results of sex-based analysis are included in Supplementary Table 1 and throughout the manuscript.
Field-collected samples	No field-collected samples were used in this study
Ethics oversight	All procedures were approved the Institutional Animal Care and Use Committee at Stanford University School of Medicine.

Note that full information on the approval of the study protocol must also be provided in the manuscript.

Plants

Seed stocks	NA
Novel plant genotypes	NA
Authentication	NA

Flow Cytometry

Plots

Confirm that:

☒ The axis labels state the marker and fluorochrome used (e.g. CD4-FITC).

☒ The axis scales are clearly visible. Include numbers along axes only for bottom left plot of group (a 'group' is an analysis of identical markers).

☒ All plots are contour plots with outliers or pseudocolor plots.

☒ A numerical value for number of cells or percentage (with statistics) is provided.

Methodology

Sample preparation	MECs were dissected from flash-frozen hemispheres from all mice that completed the random foraging electrophysiology experiments at ± 3.1 - 3.5mm lateral and 4.75 - 5.25mm posterior to bregma below 3.5mm from dorsal brain surfaces. MECs were dounce-homogenized (Wheaton, Cat. 357538) in 500 µL of EZ lysis buffer (Sigma Aldrich, NUC101) with 1 U/µL of RNase Inhibitor (Sigma Protector, 3335399001). Given this dissection approach, some nuclei from the lateral entorhinal cortex and parasubiculum may be included in this dataset. Samples were homogenized with 20 strokes each of the loose and tight pestles, and 500µL of lysis buffer was added to the samples. Samples were incubated on ice for 7 minutes before filtering through a 40 mm filter and centrifuging at 500 RCF for 5 min at 4°C. Conjugated mouse monoclonal anti-NeuN-AlexaFluor488 antibody (Millipore, Cat. MAB377X, RRID: AB_2149209) in a staining buffer (PBS with 1% BSA and 1U/µL RNase Inhibitor) was added to the tube at a final dilution of 1:250. Samples were incubated on a tube rotator for 30 min at 4°C and then spun for 5 min at 500g at 4°C. Samples were resuspended in 400µL of staining buffer with Hoechst 33342 at a final concentration of 0.01 mg/mL. Samples were then filtered through a 35mm Fluorescence-activated cell sorting (FACS) tube filter and sorted.
Instrument	A BD FACSAria Fusion with a 100 mm nozzle and with a flow rate of 1–2.5 was used.
Software	FlowJo v10.10 from BD Biosciences
Cell population abundance	48.8% of the population was non-debris, 80% of those were singlets, and 33% of those were nuclei (Hoechst+). Among nuclei, 71.4% were neurons (NeuN+). Purity of the neuronal nuclei fraction was determined by subsequent RNA sequencing of isolated nuclei.
Gating strategy	Nuclei were first gated by forward (FSC-A) and side (SSC-A) scatter, then gated for doublets with height (FSC-H) and size (FSC-A). Nuclei that were both Hoechst+ and NeuN+ were then isolated iteratively.

☒ Tick this box to confirm that a figure exemplifying the gating strategy is provided in the Supplementary Information.
